# Efficacy of therapies and interventions for repeated embryo implantation failure: a systematic review and meta-analysis

**DOI:** 10.1038/s41598-021-81439-6

**Published:** 2021-01-18

**Authors:** Andrea Busnelli, Edgardo Somigliana, Federico Cirillo, Annamaria Baggiani, Paolo Emanuele Levi-Setti

**Affiliations:** 1 Department of Gynecology, Division of Gynecology and Reproductive Medicine, Fertility Center, Humanitas Clinical and Research Center-IRCCS, Rozzano, Milan, Italy; 2 Department of Biomedical Sciences, Humanitas University, Via Rita Levi Montalcini 4, 20090 Pieve Emanuele, Milan, Italy; 3 Department of Clinical Sciences and Community Health, Università Degli Studi Di Milano, Milan, Italy; 4 Obstetrics and Gynaecology Department, Fondazione IRCCS Ca’ Granda Ospedale Maggiore Policlinico, Via M Fanti, 6, 20122 Milan, Italy

**Keywords:** Immunology, Endocrinology

## Abstract

The aim of the present systematic review and meta-analysis was to assess the effect of the different therapeutic options for repeated embryo implantation failure (RIF) on a subsequent IVF cycle outcome. Twenty-two RCTs and nineteen observational studies were included. Pooling of results showed a beneficial effect of intrauterine PBMC infusion on both CPR (RR 2.18; 95% CI 1.58–3.00; p < 0.00001; OR 2.03; 95% CI 1.22–3.36; p = 0.006) and LBR (RR 2.41; 95% CI 1.40–4.16; p = 0.002; OR 3.73; 95% CI 1.13–12.29; p = 0.03), of subcutaneous G-CSF administration on CPR (RR 2.29; 95% CI 1.58–3.31; p < 0.0001) and of intrauterine PRP infusion on CPR (RR 2.45; 95% CI 1.55–3.86; p = 0.0001). Observational studies also demonstrated a positive effect of IVIG and intrauterine hCG infusion on both CPR and LBR and of atosiban on CPR. Studies investigating intrauterine G-CSF infusion, LMWH, intravenous intralipid, hysteroscopy, blastocyst-stage ET, ZIFT, PGT-A and AH failed to observe an impact on IVF outcome. The quality of the evidence that emerged from RCTs focused on intrauterine PBMC infusion and subcutaneous G-CSF administration was moderate. For all other therapies/interventions it varied from low to very low. In conclusion, intrauterine PBMC infusion and subcutaneous G-CSF administration are the most promising therapeutic options for RIF. However, further well conducted RCTs are necessary before their introduction into clinical practice.

## Introduction

Repeated embryo implantation failure (RIF) is an extremely frustrating condition for both patients and clinicians and its treatment constitutes one of the most difficult challenges in the field of in vitro fertilization (IVF). Possible causes of RIF include wrong lifestyle habits (i.e. smoking and obesity), low quality of gametes [in particular in older women], thrombophilia, uterine factors (i.e. congenital uterine anomalies, endometrial polyps, submucosal fibroids, intrauterine adhesions) and adnexal pathologies (i.e. hydrosalpinx)^1–3^. However, in the great majority of cases, the etiology remains unknown.

### Diagnosis

The definition of RIF is controversial. Several experts consider the number of previous IVF-embryo transfer (ET) failures as a diagnostic criterion. ‘Three previous IVF-ET failed attempts’ is the most commonly used threshold ^4^. However, a minority but not negligible proportion of authors prefer a broader definition and diagnoses RIF after only two previous IVF-ET failed attempts^1^. Another school of thought suggests that the focus should be also on the number and quality of transferred embryos. According to Simon and Laufer, RIF can be defined as the failure to obtain a clinical pregnancy after three consecutive IVF attempts, in which one to two embryos of high-grade quality are transferred in each cycle^5^. Coughlan et al. proposed more stringent diagnostic criteria and defined RIF as the failure after the transfer of at least four good-quality embryos within minimum three fresh or frozen cycles under 40 years of age^6^. However, the definition of good quality embryos is subjective and the authors often do not refer to shared classification criteria.

Most of the previous meta-analyzes aimed at determining the efficacy of single therapeutic intervention for RIF included patients with at least two previous failed ET attempts. However, by applying these criteria, the rate of false positive RIF diagnosis is estimated to be considerable [at least 46%]^7^ and, as a consequence, the studied population probably included a significant proportion of patients without a real obstacle to conception but who had not yet succeeded just because of statistical misfortune. Evidence about efficacy of therapeutic interventions deriving from meta-analyzes conducted with these assumptions cannot therefore be considered completely reliable.

In the present systematic review and meta-analysis, we defined RIF as the failure to obtain a clinical pregnancy after at least three ET attempts. By using this threshold, the risk of false positive diagnosis is significantly lower^7^. Importantly, these diagnostic criteria also exclude elements of subjectivity and are therefore easily replicable in any clinical setting.

### Therapies and interventions

Proposed therapies and interventions for RIF can be grouped in four categories:

1. *Uterine interventions* (e.g. intentional endometrial injury; hysteroscopy; endometrial sampling for histology and microbiological investigations and endometritis treatment; atosiban administration; copper intrauterine device placement)^8–12^;
2. *Laboratory and procedural technologies and interventions* (i.e. sequential ET [i.e. sequential ET on day 2/3 and on day 5); ET medium enriched with hyaluronic acid; autologous embryo-cumulus cells co-culture; intracytoplasmic morphologically selected sperm injection (IMSI); blastocyst stage ET; zygote intrafallopian tube transfer (ZIFT); assisted hatching (AH); preimplantation genetic testing for aneuploidies (PGT-A))^13–20^;
3. *Immunomodulatory therapies* (e.g. intravenous immunoglobulin (IVIG); intrauterine peripheral blood mononuclear cell (PBMC) infusion; tacrolimus; subcutaneous or intrauterine granulocyte colony stimulating factor (G-CSF) administration; intrauterine autologous platelet-rich plasma (PRP) infusion; intravenous intralipid; intrauterine human chorionic gonadotropin (hCG) injection; low-molecular-weight heparin (LMWH); aspirin; prednisolone)^21–28^;
4. Treatments enhancing endometrial receptivity or technologies aimed at identifying the endometrial window of implantation (WOI) (e.g. intramuscular growth hormone (GH); vaginal sildenafil; endometrial receptivity array (ERA))^29–33^.

In most cases, the abovementioned therapeutic interventions are promising. However, clinicians can hardly orient themselves toward such a plethora of options with often unproven efficacy^2^.

### Aim

Considering the methodological weaknesses of the previous contributions and the uncertainties about the preferred treatment strategies, we conducted the present systematic review and meta-analysis with the aim to assess the effect of the different therapies and interventions for RIF on the subsequent IVF cycle outcomes.

## Materials and methods

This literature overview was reported according to the PRISMA guidelines for systematic reviews^34,35^ and the meta-analysis was conducted according to the MOOSE guidelines^36^. Since published de-identified data were used, this study was exempt from institutional review board approval.

### Sources and study selection

The present systematic review and meta-analysis was restricted to published research articles that investigated the effect of all proposed therapies and interventions for RIF on the subsequent IVF cycle outcomes. Primary outcomes were Live Birth Rate (LBR) per patient and Clinical Pregnancy Rate (CPR) per patient. “Live birth” was defined as the delivery of one or more living infants. “Clinical pregnancy” was defined as the presence of one or more intrauterine gestational sacs on transvaginal ultrasound or other definitive clinical signs^37^. Secondary outcomes were implantation rate (IR) per embryo, multiple pregnancy rate (MPR) per patient and miscarriage rate (MR) per patient. “Implantation rate” was defined as the number of gestational sacs on transvaginal ultrasound divided by the number of embryos transferred. “Multiple pregnancy” was defined as the presence of two or more intrauterine embryos on transvaginal ultrasound. “Miscarriage” was defined as fetal loss before 20 weeks’ gestation^37^.

We systematically searched Pubmed, MEDLINE, Embase and Scopus, from database inception to May 13th, 2020. Searches were limited to studies in humans. A first search was conducted using the following terms: ‘therapy’ OR ‘intervention’ OR ‘treatment’ AND ‘implantation failure’ OR ‘repeated implantation failure’ OR ‘recurrent implantation failure’ OR ‘RIF’. A second search was carried out by combining each therapy or intervention emerged from the first search (i.e. endometrial injury; hysteroscopy; endometrial sampling for histology and microbiological investigations and endometritis treatment; atosiban; copper intrauterine device placement; sequential embryo transfer; embryo transfer medium enriched with hyaluronic acid; autologous embryo-cumulus cells co-culture; intracytoplasmic morphologically selected sperm injection; blastocyct stage embryo transfer; zygote intrafallopian tube transfer; assisted hatching; preimplantation genetic testing for aneuploidies; intravenous immunoglobulin; intrauterine administration of peripheral blood mononuclear cell; tacrolimus; subcutaneous administration of granulocyte colony stimulating factor; intrauterine infusion of autologous platelet-rich plasma; intravenous intralipid infusion; human chorionic gonadotropin; low-molecular-weight heparin; aspirin; growth hormone; corticosteroids; vaginal sildenafil; endometrial receptivity array) AND ‘implantation failure’ OR ‘repeated implantation failure’ OR ‘recurrent implantation failure’ OR ‘RIF’.

Studies could be included only if: (1) RIF was defined as the failure to obtain a clinical pregnancy after at least three ET attempts, (2) the included patients were investigated in order to exclude possible known causes of RIF, (3) they compared IVF outcomes between treated RIF patients and untreated RIF patients.

We considered eligible for inclusion published randomized controlled trials (RCTs), cohort and case control studies. Reference lists of all pertinent articles, systematic review and meta-analysis on the argument were systematically reviewed with the aim of identifying further studies that could be evaluated for inclusion. No attempt was made to identify unpublished studies.

Two authors (A.B. and P.E.L.S.) independently screened title and abstract of all articles to exclude studies deemed irrelevant. In case of opinion discrepancy, studies were discussed with two other investigators (F.C. and A.Ba.). Reports were classified according to the study design into RCTs, case–control studies, prospective and retrospective cohort studies.

### Risk of bias and quality assessment

Two authors (A.B. and E.S.) independently assessed the included studies for risks of bias using the Cochrane 'Risk of bias' assessment tool^38^ for randomized clinical trials (RCTs) and the ROBINS-I tool^39^ for observational studies.

We assessed the quality of the evidence using GRADE criteria: risk of bias, consistency of effect, indirectness, imprecision and publication bias^40^. Two review authors (P.E.L.S. and A.Ba.) working independently made judgements about evidence quality [high, moderate, low or very low], with disagreements resolved by discussion. We justified, documented, and incorporated our judgements into the reporting of results for each outcome.

### Data extraction and analysis

Three authors (A.B., E.S. and F.C.) independently evaluated all articles and extrapolated the data on standardized forms. A final abstraction form was compiled from the three evaluation forms, after resolution of all the discrepancies among reviewers through a discussion with the two remaining authors.

The year of publication, location, study design, study period, criteria used to define RIF, investigations performed to exclude possible known causes of RIF, investigated therapy or intervention for RIF, primary and secondary outcomes were recorded.

Study outcomes were expressed using risk ratio (RR) with 95% confidence interval (95% CI) for RCTs and odds ratio (OR) with 95% CI for observational studies.

Risk estimates greater than 1 indicate an increased risk of the defined outcome; risk estimates less than 1 indicate a decreased risk of the defined outcome. We assessed statistical significance using 95%CI: if the 95%CI did not include the neutral value 1, we considered the risk statistically significant^41,42^. The inconsistency of the studies' results was measured using Cochrane Q and the I^2^ statistic^38^. Negative values of I^2^ are set equal to 0 so that I^2^ lies between 0 and 100%. According to the Cochrane Handbook for Systematic Reviews of Intervention, an I^2^ value of 0 indicates no observed heterogeneity, whereas I^2^ values from 30 to 60% may represent moderate heterogeneity, I^2^ values from 50 to 90% may represent substantial heterogeneity, and I^2^ values from 75 to 100% represent considerable heterogeneity^38^. If the I^2^ values indicated moderate, substantial, or considerable heterogeneity, we conducted sensitivity analyses to verify whether any one of the included studies unduly influenced the pooled effect size.

The risk estimates were combined in a meta-analysis using a fixed effects model when the heterogeneity found among the studies was absent to moderate (0% ≤ I^2^ < 30%). When heterogeneity was moderate, substantial, or considerable (I^2^ ≥ 30%), we used the DerSimonian and Laird method^43,44^ for a random-effects model^45^. Funnel plots, which graph RR/OR on a log scale (effect) against standard error of log-RR/OR (precision), were generated and visually inspected for asymmetry to determine whether the included studies were non representative of the body of possible studies on the subject (as could result from a small-study effect or other biases, such as publication and poor-quality bias). The approach by Egger et al. was used to test the significance of funnel plot asymmetry^45^. All analyses were performed using Review Manager version 5.3 (Nordic Cochrane Centre, Cochrane Collaboration).

## Results

### Results of search and description of studies

**Figure 1:**
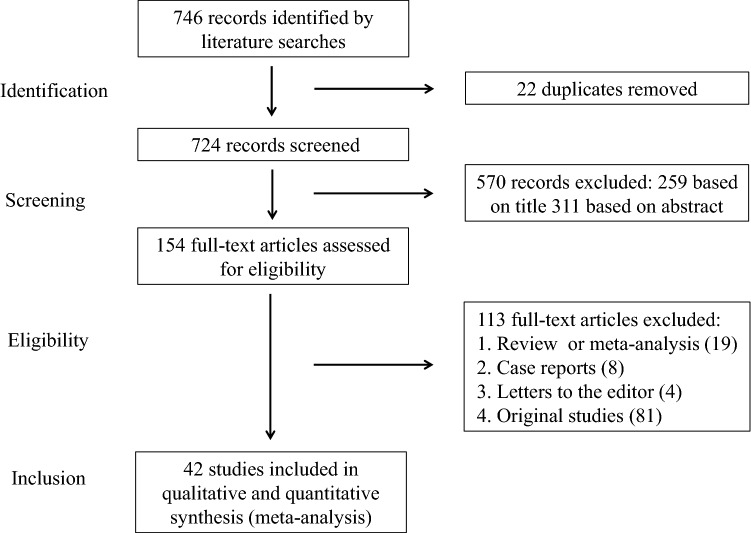
Study selection.

Figure 1 summarizes the process of literature identification and selection of studies. Our literature searches yielded 746 studies, from which 22 duplicates were removed. After a review of the titles and abstracts, 154 studies were identified as potentially eligible for inclusion. After a full review, we excluded 19 systematic reviews or meta-analysis^2,5,22,23,37,46–59^, 8 case reports^60–67^, 4 letters to the editor^68–71^ and 81 original studies [references and reasons for exclusion are reported in Table 1]. Data on the efficacy of therapies and interventions for RIF were extracted from the remaining 42 articles^8,12,13,18,20,21,24,27,28,31,75,140–170^. Included studies investigated uterine interventions, laboratory and procedural technologies and interventions and immunomodulatory therapies. Details of the characteristics of the selected studies are shown in Table 2. Seven of the included studies were case–control studies, 12 were prospective cohort studies and 22 were RCTs. Therapies and interventions that could be pooled included subcutaneous or intrauterine G-CSF administration, sequential ET, intravenous intralipid infusion, endometrial injury, subcutaneous LMWH, hysteroscopy, PGT-A, atosiban, IVIG administration, intrauterine hCG injection, blastocyst stage ET, ZIFT, intrauterine PBMC infusion, AH and intrauterine PRP infusion.

**Table 1:** Reasons for exclusion of observational studies.

References	Therapy/intervention	Reason for exclusion
Aghajanzadeh et al.^26^	Intrauterine PRP	Exclusion of possible known cause of RIF not mentioned
Ahmadi et al.^72^	Sirolimus	Control arm not adequate
Ahmadi et al.^73^	IVIG	RIF criteria not clearly reported
Akhtar et al.^29^	Aspirin and Heparin	RIF criteria: one or more unsuccessful IVF cycle
Al Turki^74^	Hysteroscopy	RIF criteria: two previous IVF failures
Almog et al.^75^	Interval double transfer	Exclusion of possible known causes of RIF not mentioned
Aslan et al.^76^	ZIFT	Exclusion of possible known causes of RIF not mentioned
Altmäe et al.^30^	Growth hormone	RIF criteria: two or more previous IVF failures
Arefi et al.^77^	G-CSF	RIF criteria: confusion on the number of previous failed IVF attempts
Bar et al.^78^	Endometrial scratching	RIF criteria: at least two failed IVF cycles
Barash et al.^79^	Endometrial injury	RIF criteria: one or more previous IVF failures
Barrenetxea et al.^80^	Blastocyst transfer at day 6	Exclusion of possible known causes of RIF not mentioned
Benkhalifa et al.^15^	Autologous embryo–cumulus cells co-culture	Exclusion of possible known causes of RIF not mentioned
Chao et al.^81^	Assisted hatching	RIF criteria: two or more previous IVF failures
Cicinelli et al.^9^	Hysteroscopy and endometrial sampling for histology and microbiological investigations	Absence of an adequate control arm/group
Debrock et al.^82^	Quarter Laser-Assisted Zona Thinning	Exclusion of possible known causes of RIF not mentioned
Delaroche et al.^16^	IMSI	RIF criteria and inadequate control group
Dunne and Taylor 2014^83^	Endometrial injury	RIF criteria: one or more previous IVF failures
Edirisinghe et al.^19^	Assisted hatching	Exclusion of possible known cause of RIF not mentioned
Eftekhar et al.^84^	G-CSF	RIF criteria: two or more episodes of implantation failure
El Khattabi et al.^85^	IMSI	RIF criteria: at least two implantation failures after transfers of good-quality embryos
Friedler et al.^86^	Embryo transfer medium enriched with hyaluronan	Exclusion of not all possible known causes of RIF not mentioned
Fu et al.^14^	Hyaluronic acid–enriched transfer medium	RIF criteria
Fwzy and El-Refaeey^87^	LMWH and prednisolone	RIF criteria: history of previously failed one or two implantations at the same center
Gao et al.^10^	Hysteroscopy	RIF criteria: two or more consecutive ET failures with at least one good-quality cleavage embryos on day 3 in each ET
Gatimel et al.^88^	IMSI	RIF criteria: two previous IVF failures
Gianaroli et al.^89^	PGT-A	RIF criteria: two or more previous IVF failures
Gibreel et al.^90^	Endometrial injury	RIF criteria: at least one previous failed IVF cycle
Hamdi et al.^91^	LMWH	RIF criteria: at least 2 cases of implantation failure with fresh embryo with good grades
Hayashi et al.^92^	Endometrial injury	Exclusion of possible known causes of RIF not mentioned
Heilmann et al.^93^	IVIG-Treatment	Exclusion of possible known causes of RIF not mentioned
Hiraoka et al.^94^	Assisted hatching	RIF criteria: two or more previous IVF failures
Hosseini et al.^95^	Hysteroscopy	RIF criteria: ≥ two ART cycles with fresh and good quality and quantity embryos
Huang et al.^96^	Endometrial injury	RIF criteria: two or more previous IVF failures
Inal et al.^97^	Endometrial injury	RIF criteria: one or more cycles of IVF and ET
Jayot et al.^98^	Coculture of embryos on homologous endometrial cells	Exclusion of possible known causes of RIF not mentioned
Jelinkova et al.^99^	Assisted hatching	RIF criteria: two or more previous IVF failures
Johnston-MacAnanny et al.^100^	Endometritis treatment	RIF criteria: at least two failed cycles of IVF-ET
Kanazawa et al.^101^	Endometrial injury	RIF criteria: two or more previous FET failures
Kanyo et al.^102^	Assisted hatching	Inclusion criteria: maximum three previous failed IVF cycles
Karabulut et al.^103^	IMSI	Exclusion of possible known causes of RIF not mentioned
Karacan et al.^104^	Blastocyst transfer	RIF criteria: at least two previously failed IVF attempts
Karimzadeh et al.^105^	Endometrial injury	RIF criteria: at least 2 unsuccessful cycles of IVF-ET
Kitaya et al.^106^	Endometritis treatment	RIF criteria
Lambers et al.^107^	Low-dose aspirin	RIF criteria: at least one previous IVF failed conception
Lee et al.^108^	PGT-A	Exclusion of possible known causes of RIF not mentioned
Lee et al.^109^	Assisted hatching	RIF criteria: two or more episodes of implantation failure
Lodigiani et al.^110^	LMWH	RIF criteria: two or more episodes of implantation failure
Loutradis et al.^111^	Sequential ET	Exclusion of possible known causes of RIF not mentioned
Lu et al.^112^	Assisted hatching	RIF criteria: more than one failed IVF treatment
Madhavan et al.^113^	Intrauterine PRP	RIF criteria: at least one previous failed FET
Mak et al.^114^	Endometrial injury	Numerator not reported
Mao et al.^11^	Copper intrauterine device placement	RIF criteria: two or more previous implantation failures
Moini et al.^115^	Vaginal sildenafil	RIF criteria: two prior consecutive failed IVF/ICSI attempts
Munné et al.^116^	PGT-A	Inclusion criteria: history of two or fewer prior implantation failures following IVF
Murat Seval et al.^117^	Endometrial injury	RIF criteria: absence of implantation after two consecutive cycles of IVF, ICSI, or frozen embryo replacement cycles
Nakagawa et al.^25^	Th1/Th2 ratio assessment and tacrolimus administration	Absence of an adequate control arm/group
Narvekar et al.^118^	Endometrial injury	RIF criteria: at least one previous failed IVF-ET/ICSI cycle
Ng et al.^119^	Atosiban	Exclusion criteria: three or more previous IVF failures
Oliveira et al.^120^	IMSI	RIF criteria: at least two prior unsuccessful ICSI cycles
Petersen et al.^121^	Assisted hatching	RIF criteria: two or more episodes of implantation failure
Qublan et al.^122^	LMWH	Patients with thrombophilia included
Rama Raju et al.^123^	Hysteroscopy	RIF criteria: two or more previous failed IVF cycles
Ruiz-Alonso et al.^32^	Endometrial receptivity array	Control group not adequate
Shahrokh Tehraninejad et al.^124^	Endometrial injury	RIF criteria: at least two failure of IVF/ICSI cycles
Shalom-Paz et al.^125^	IMSI	Control group not adequate
Shohayeb and El-Khayat^126^	Endometrial injury	RIF criteria: history of two or more failed ICSI cycles
Singh et al.^127^	Endometrial injury	RIF criteria: two or more IVF failed attempts
Singh et al.^128^	Intravenous intralipid	RIF criteria: at least one previous implantation failure
Siristatidis et al.^129^	Endometrial injury	RIF criteria: failure of implantation in at least two IVF attempts
Siristatidis et al.^38^	LMWH and prednisolone	RIF criteria: at least two failed fresh IVF/ICSI cycles
Stein et al.^130^	Assisted hatching	Exclusion of possible known cause of RIF not mentioned
Tan et al.^131^	Endometrial receptivity array	RIF criteria: two or more previous implantation failures
Tersoglio et al.^132^	Endometritis treatment	RIF criteria: absence of implantation after two or more cycles of IVF / ICSI or cryotransfer
Tk et al.^133^	Endometrial injury	RIF criteria: at least one previous IVF failed cycle
Tumanyan et al.^134^	Endometrial injury	RIF criteria: failed implantation after transfer of seven or more top quality day 3 embryos or three blastocysts
Valojerdi et al.^135^	Assisted hatching	RIF criteria: two or more previous failed IVF cycles
Volovsky et al.^136^	Intrauterine infusion of HCG	Exclusion of possible known cause of RIF not mentioned
Yang et al.^137^	Endometritis treatment	Exclusion of possible known cause of RIF not mentioned
Yeung et al.^138^	Endometrial injury	RIF criteria: one previous implantation failure
Zhang et al.^139^	Fertiloscopy	RIF criteria: at least two failed IVF-ET cycles

*PRP* platelet rich plasma, *RIF* repeated implantation failure, *IVIG* intravenous immunoglobulin, *IVF* in vitro fertilization, *IMSI* intracytoplasmic morphologically selected sperm injection, *IVF* in vitro fertilization, *ET* embryo transfer, *G-CSF* granulocyte-colony stimulating factor, *ZIFT* zygote intrafallopian transfer, *LMWH* low molecular weight heparin, *hCG* human chorionic gonadotropin, *PGT-A* preimplantation genetic testing for aneuploidy.

**Table 2:** Characteristics of the included studies.

Study	Country	Design	Age of included women	RIF diagnostic criteria	COH protocol	Therapy/intervention	No. of patients	Outcomes
Aleyasin et al.^140^	Iran	Prospective randomized open-label controlled trial	< 40 years	Failure of implantation in at least three consecutive IVF attempts, in which three embryos of high-grade quality are transferred in each cycle	Long Protocol	A single dose of 300 μg G-CSF (Neupogen; Roche) administered subcutaneously 1 h before the embryo transfer	112	IR; CPR
Almog et al.^75^	Israel	Retrospective case control study	34.3 ± 0.7 years (cases); 34.7 ± 0.1 years (controls)	A minimum of three previous IVF/ET failures	Short agonist protocol	Sequential embryo transfer	131	CPR; MPR
Al-Zebeidi et al.^27^	Saudi Arabia	Randomized controlled trial	< 42 years	Failure to achieve a pregnancy despite more than three times of ICSI cycles	Long or antagonist protocol	Intralipid 20% 100 ml diluted in 500 ml normal saline for intravenous infusion	142	CPR; MR; LBR
Baum et al.^8^	Israel	Randomized controlled trial	≤ 41 years	Three or more unsuccessful cycles of IVF with good ovarian response in previous cycles	Long agonist, antagonist protocol and short agonist protocol	Endometrial injury: endometrial biopsies performed using a pipelle curette on days 9–12 and 21–24 of the menstrual cycle preceding IVF treatment	36	IR; CPR; MR; LBR
Berker et al.^31^	Turkey	Prospective quasi-randomized controlled study	≤ 44 years (cases); ≤ 46 years (controls)	Three or more consecutive failed cycles of ICSI	Long agonist, antagonist protocol and short agonist protocol	LMWH at a standard dose of 40 mg/0.4 mL per day starting on the day of oocyte retrieval to the 12th week of pregnancy	91	CPR; LBR
Blockeel et al.^141^	Belgium	Randomized controlled trial	< 37 years	Three or more failed IVF/ICSI cycles with embryo of good morphological quality	Long agonist, antagonist protocol and short agonist protocol	PGT-A	139	CPR; LBR
Davari-tanha et al.^142^	Iran	Randomized double blind placebo controlled clinical trial	< 40 years	History of three times implantation failure when there was history of transferring at least four good quality embryos	Not reported in details	At the time of oocyte retrieval one ml of G-CSF (Nupogen (300 μg/ml, Filgrastim; Amgen)) was administered by a Trans cervical Cook catheter for embryo transfer slowly into uterine cavity	100	IR; CPR; MR
El-Thouky et al.^143^	United Kingdom, Belgium, Italy, Czech Republic	Multicentre, randomised controlled trial	< 38 years	At least three previous unsuccessful IVF treatment cycles	Not reported in details	Hysteroscopy	330	LBR
Fang et al.^144^	China	Retrospective case control study	≤ 40 years	Three or more IVF cycle failures	Long protocol	Sequential embryo transfer	180	IR; CPR; MPR
Greco et al.^145^	Italy	Retrospective case control study	< 36 years	History of 3–9 (mean 4.9) implantation failures in previous IVF attempts	Long protocol	PGT-A	76	IR; CPR; MR
Gürgan et al.^146^	Turkey	Randomized controlled trial	< 40 years	The failure to achieve a clinical pregnancy after the transfer of at least four good-quality embryos in a minimum of three fresh or frozen cycles	Standard long agonist or antagonist protocols	Hysteroscopic endometrial injury: endometrial injury on the 10th–12th day of the late follicular phase in the preceding cycle through office hysteroscopy	305	IR; CPR; LBR
He et al.^12^	China	Prospective cohort study	≤ 45 years	Three or more ET failures	Endometrial preparation (natural cycle, HRT) for frozen embryo transfer	Atosiban (Tractocile; Ferring Pharmaceuticals) as an i.v. bolus of 6.75 mg at about 30 min prior before ET	88	IR; CPR; MR
Ho et al.^21^	Taiwan	Retrospective case control study	35.4 ± 4.7 years (cases) and 36.5 ± 4.4 years (controls)	Three or more failures of IVF–embryo transfer therapy with at least two good embryos transferred each session	Long protocol	First dosae of IVIG (24 g TBSF human immunoglobulin; CSL Limited, Australia) on day 8 of the stimulating cycle. If a viable pregnancy was confirmed, IVIG was continued in the 4, 6, and 10^th^ weeks of gestation age (a total dose of 96 g)	283	IR; CPR; LBR
Huang et al.^28^	China	Retrospective case control study	≤ 38 years	Three or more ET failures	Endometrial preparation (natural cycle, letrozole induction, HRT) for frozen-thawed blastocyst transfer	1000 IU of hCG via an intrauterine injection 3 days before the ET	179	CPR
Kalem et al.^147^	Turkey	Randomized controlled trial	< 40 years	Failure to achieve a clinical pregnancy after the transfer of at least four good-quality embryos in a minimum of three fresh or frozen cycles	Long or antagonist protocol	Administration of 30 mIU of Leucostim (Filgrastim [G-CSF] 30mIU/mL; DEM Medical, Dong-A; South Korea) through infusion into the endometrial cavity	157	CPR; MR; LBR
Kim et al.^148^	South Korea	Randomized controlled trial	≤ 40 years	Failure of good quality embryos to implant after at least three cycles of IVF/ICSI	GnRH antagonist protocol	G-CSF at a dose of 100 mcg was administered subcutaneously on the day of ET and the fourth day after ET	82	CPR
Levitas et al.^149^	Israel	Prospective randomized study	< 37 years	At least three previous IVF/ET cycles failures	Long protocol	Blastocyst-stage embryo transfer	54	IR; CPR; LBR
Levran et al.^18^	Israel	Case control study	31.1 ± 5.4 years (cases); 30.6 ± 5.3 years (controls)	At least three failures of implantation in IVF-ET cycles in which at least three embryos were placed per transfer	GnRH agonist protocol	ZIFT 24 -26 h after oocyte retrieval using a three-puncture laparoscopy method	140	IR; CPR; MR
Levran et al.^150^	Israel	Prospective nonrandomized study	≤ 43 years	A minimum of three previous failed IVF-ET attempts, excluding frozen-thawed embryo transfers	Long or short GnRH agonist protocol	ZIFT 24–48 h after oocyte retrieval, and zygotes were transferred into one tube via laparoscopy	64	IR; CPR; LBR
Li et al.^24^	China	Prospective patient’s treatment preference	30.83 ± 4.10 years (cases); 30.51 ± 4.08 years (controls)	Three or more failures of IVF-ET therapy	Endometrial preparation (natural cycle, HRT) for frozen-thawed embryo transfer	Intrauterine administration of cultured PBMC (1–2 × 107cells/200 µl) one day before frozen/thawed embryo transfer using embryo transfer catheter	216	CPR; LBR
Liu et al.^151^	China	Prospective cohort study	≤ 45 years	Implantation failure after three or ET of high quality embryos	Endometrial preparation (natural cycle, HRT) for frozen-thawed blastocyst transfer	Intrauterine injection of 500 IU of hCG 3 days before embryo transfer	305	IR; CPR; MR; LBR
Matsumoto et al.^152^	Japan	Prospective cohort study	< 40 years	At least three unsuccessful ET	Endometrial preparation (HRT) for frozen-thawed blastocyst transfer	Endometrial injury: scratching was performed once during the luteal phase of the cycle preceding the one that was used for the embryo transfer	77	CPR
Rufas-Sapir et al.^153^	Israel	Randomized controlled trial	≤ 41 years	Three or more failures of IVF-ET therapy	Not reported	AH	207	CPR
Madkour et al.^154^	Morocco	Randomized controlled trial	< 40 years	Three or more previous IVF failures	GnRH antagonist	Intrauterine administration of PBMC prior to fresh embryo transfer	27	CPR
Nazari et al.^155^	Iran	Randomized controlled trial	< 40 years	Three or more ET failures with high-quality embryos	Endometrial preparation (HRT) for FET	Intrauterine infusion of autologous PRP carried out 48 h before ET	97	CPR
Nobijari et al.^156^	Iran	Randomized controlled trial	36.17 ± 4.60 years (cases); 35.16 ± 5.11 years (controls)	Three or more previous IVF failures	Endometrial preparation (HRT) for FET	Intrauterine administration of PBMC 2 days before the scheduled embryo transfer	138	CPR
Okitsu et al.^157^	Japan	Prospective patient’s treatment preference study	37.4 ± 5.33 years (cases); 38.3 ± 4.20 years (controls)	Failed to conceive after at least 3 IVF-ET sessions	Long or antagonist protocol	Intrauterine administration of autologous PBMC	55	IR; CPR; LBR
Primi et al.^158^	Switzerland, Germany, France, Spain	Case control study	≤ 45 years	Three previous nidation failures of fresh embryos, including each time the transfer of at least two embryos of good quality	Not reported in details	AH	74	CPR; MR; LBR
Raziel et al.^159^	Israel	Prospective patient's treatment preference study	< 40 years	Four or more ET of fresh embryos and the cumulative transfer of at least 12 fresh embryos without the achievement of a clinical pregnancy	Long protocol	Endometrial injury: endometrial biopsy performed on days 21 and 26 of the spontaneous cycle	117	CPR
Rubio et al.^20^	Spain	Randomized controlled trial	< 40 years	Three or more previous IVF/ICSI attempts and transfer of good-quality embryos	Not reported in details	PGT-A	91	CPR; MR; LBR
Sato et al.^160^	Japan	Multi-centre prospective pilot study	≤ 42 years	History of three or more implantation failures after IVF-ET treatment	Long or short agonist or antagonist protocol	PGT-A	92	BPR; CPR; LBR
Scarpellini and Sbracia^161^	Italy	Randomized controlled trial	< 39 years	At least three previous failed IVF attempts where at least 7 good embryos were transferred	Not reported	Subcutaneous G-CSF 60 mg/daily from the day of transfer to the day of pregnancy test, and if it was positive the treatment was continued for other 40 days	109	CPR
Scarpellini and Sbracia^162^	Italy	Randomized controlled trial	< 39 years	Three previous failed IVF attempts with 8 good embryos were transferred	Not reported	Subcutaneous G-CSF 60mcg/daily from the day of transfer to the day of pregnancy test	69	CPR
Shahrokh Tehraninejad et al.^13^	Iran	Randomized controlled trial	≤ 40 years	Three previous IVF failures	Long protocol	Sequential transfer	120	BPR; CPR; MPR
Shahrokh Tehraninejad et al.^163^	Iran	Prospective study	≤ 43 years	A minimum of three previous failed IVF-ET cycles	Long protocol	ZIFT performed 24 h after oocyte retrieval with the use of a three- puncture laparoscopy method	250	BPR; CPR; MR;LBR
Olesen et al.^164^	Denmark	Randomized controlled trial	≤ 40 years	Three or more previous failed implantations	GnRH-antagonist protocol	Endometrial injury: Scratching was performed, using a Pipelle de Cornier (Laboratoires Prodimed) in the luteal phase before ovarian stimulation at cycle day 18–22 for the intervention group	117	IR; CPR; MR; LBR
Urman et al.^165^	Turkey	Randomized open-labeled pilot trial	≤ 38 years	Three or more previously failed fresh embryo transfer cycles	Long protocol	LMWH (Enoxaparin Sodium, Clexane, Aventis Pharma) at a dose of 1 mg/kg/day starting on the day after oocyte retrieval; LMWH was continued up to the 12th week of pregnancy if the test was positive	71	CPR; LBR
Xiong et al.^166^	China	Randomized controlled trial	34.89 ± 2.49 years (cases); 35.05 ± 2.79 years (controls)	Three or more previous IVF failures	Not reported	LMWH IU/day were administered from ET, until detection of the fetal heart	147	CPR
Yakin et al.^167^	Turkey	Prospective nonrandomized parallel group study	≤ 38 years	History of at least three previously failed fresh embryo transfer cycles	Long protocol	PGT-A	140	CPR; LBR
Yoshioka et al.^168^	Japan	Prospective patient’s treatment preference study	37.5 ± 4.4 years (cases); 36.6 ± 4.4 years (controls)	Four or more failures of IVF-ET cycles	Long protocol	Intrauterine administration of PBMC on day 2 of embryo culture	35	IR; CPR; LBR
Yu et al.^169^	China	Randomized controlled trial	< 35 years	Three or more failed IVF-ET sessions	Endometrial preparation (natural cycle, HRT) for frozen-thawed blastocyst transfer	Intrauterine administration of autologous PBMC activated by hCG in vitro 1 day before ET	198	CPR; LBR
Zamaniyan et al.^170^	Iran	Randomized controlled trial	≤ 40 years	Three or more ET failures	Endometrial preparation (HRT) for FET	Intrauterine infusion of autologous PRP carried out 48 h before ET	98	CPR

*IVF* in vitro fertilization, *RIF* repeated implantation failure, *COH* controlled ovarian hyper stimulation, *ICSI* intracytoplasmic sperm injection, *ET* embryo transfer, *HRT* hormone replacement therapy, *FET* frozen embryo transfer, *G-CSF* granulocyte-colony stimulating factor, *LMWH* low molecular weight heparin, *PBMC* peripheral blood mononuclear cells, *AH* assisted hatching, *PGT-A* preimplantation genetic testing for aneuploidy, *IVIG* intravenous immunoglobulin, *PRP* platelet rich plasma, *hCG* human chorionic gonadotropin, *ZIFT* zygote intrafallopian transfer, *IR* implantation rate, *CPR* clinical pregnancy rate, *MR* miscarriage rate, *MPR* multiple pregnancy rate, *LBR* live birth rate.

### Risk of bias and quality assessment results

**Figure 2:**
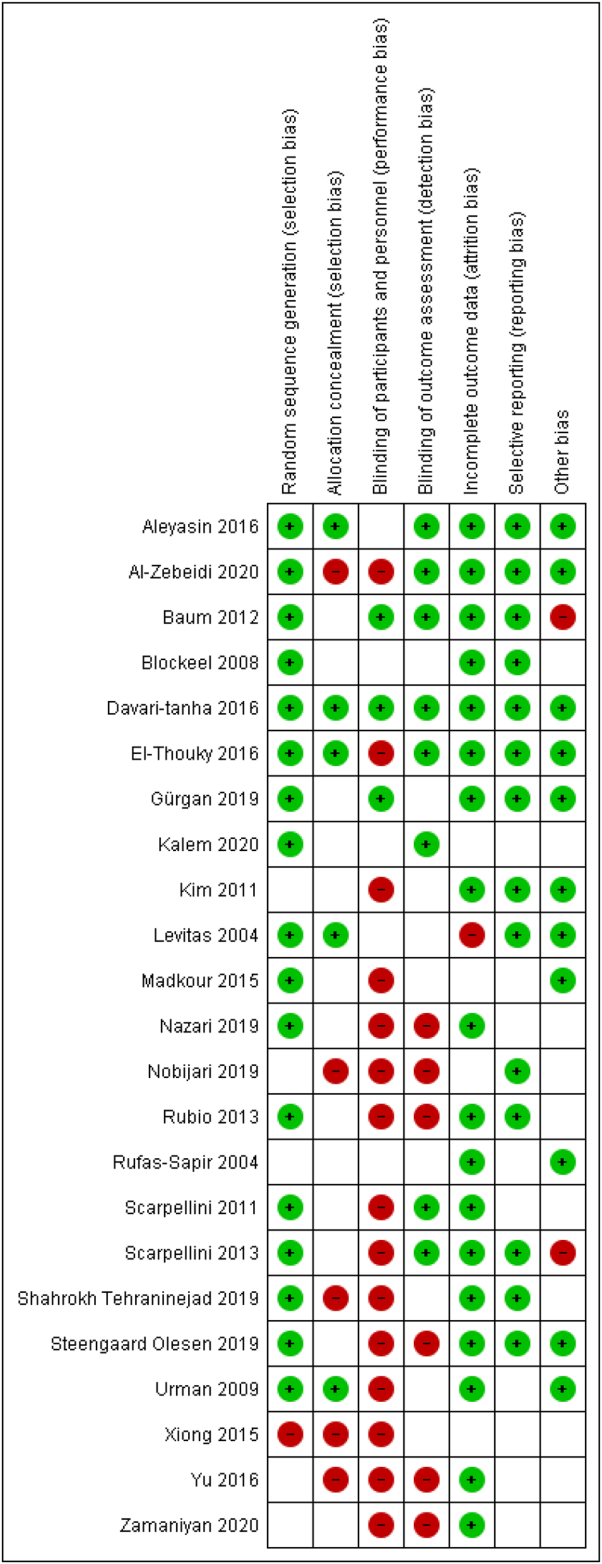
Risk of bias summary: review authors' judgements about each risk of bias item for each included randomized controlled trial (RCT).

Results obtained from the risk of bias assessment for RCTs and for observational studies are summarized in Fig. 2 and Table 3 respectively. The quality of the evidence for each single therapy/intervention is described in the ‘Synthesis of results’ section and summarized in Table 4.

**Table 3:** Assessment of risk of bias of non randomized studies according to the ROBINS-I tool.

References	Preintervention		At intervention	Post intervention				Overall risk of bias
	Bias due to confounding	Bias in selection of participants into the study	Bias in classification of interventions	Bias due to deviations from interventions	Bias due to missing data	Bias in measurement of outcomes	Bias in selection of the reported result	Low/moderate/serious/critical
Almog et al.^75^	Low	Moderate	Moderate	Moderate	Low	Moderate	Low	Moderate
Berker et al.^31^	Low	Low	Low	Moderate	Low	Low	Low	Moderate
Fang et al.^144^	Serious	Moderate	Moderate	Low	Low	Moderate	Low	Serious
Greco et al.^145^	Moderate	Moderate	Low	Moderate	Low	Low	Low	Moderate
He et al.^12^	Moderate	Moderate	Low	Low	Low	Low	Moderate	Moderate
Ho et al.^21^	Moderate	Low	Moderate	Low	Low	Moderate	Low	Moderate
Huang et al.^48^	Low	Low	Moderate	Low	Serious	Moderate	Moderate	Serious
Levran et al.^18^	Moderate	Moderate	Low	Low	Low	Moderate	Low	Moderate
Levran et al.^150^	Moderate	Low	Low	Low	Low	Moderate	Low	Moderate
Li et al.^24^	Moderate	Moderate	Moderate	Moderate	Low	Low	Low	Moderate
Matsumoto et al.^152^	Low	Low	Low	Low	Low	Low	Low	Low
Okitsu et al.^157^	Moderate	Moderate	Moderate	Low	Low	Low	Low	Moderate
Primi et al.^158^	Moderate	Moderate	Low	Low	Low	Low	Low	Moderate
Raziel et al.^159^	Moderate	Moderate	Moderate	Low	Low	Low	Low	Moderate
Sato et al.^160^	Low	Low	Low	Low	Low	Low	Low	Low
Shahrokh Tehraninejad et al.^163^	Moderate	Moderate	Low	Low	Low	Moderate	Low	Moderate
Yakin et al.^167^	Low	Low	Low	Low	Low	Low	Low	Low
Yoshioka et al.^168^	Low	Moderate	Moderate	Low	Low	Low	Low	Moderate

**Table 4:** Summary of findings and certainty of the evidence.

Therapy/intervention	Outcome	RCTs/Observational studies	Number of studies	Number of participants	Effect (95% CI)	GRADE score (RCTs = + 4; Observational studies = + 2)							GRADE quality of the evidence
						Quality	Consistency	Directness	Precision	Publication bias	Upgrading	Total score	
Intrauterine G-CSF	LBR	RCTs	1	157	RR 0.84 (0.41–1.73)	− 1	0	0	− 1	0	0	2	Low
	CPR	RCTs	2	257	RR 1.53 (1.00–2.33)	− 1	0	0	− 1	0	0	2	Low
	IR	RCTs	1	100	RR 2.28 (0.90–5.74)	− 1	0	0	− 1	0	0	2	Low
	MR	RCTs	1	157	RR 3.20 (0.69–14.93)	− 1	0	0	− 1	0	0	2	Low
Subcutaneous G-CSF	CPR	RCTs	4	333	RR 2.29 (1.58–3.31)	− 1	0	0	− 1	0	+ 1 (magnitude)	3	Moderate
	IR	RCTs	1	112	RR 2.94 (1.24–5.01)	− 1	0	0	− 1	0	0	2	Low
Sequential ET	CPR	RCTs	1	120	RR 1.04 (0.67–1.63)	− 2	0	0	− 1	0	0	1	Very low
	CPR	Observational studies	2	282	OR 2.64 (1.56–4.47)	− 1	0	0	0	0	0	1	Very low
	IR	Observational studies	1	151	OR 2.95 (1.65–5.27)	− 1	0	0	0	0	0	1	Very low
Intralipid	LBR	RCTs	1	142	RR 1.30 (0.61–2.77)	− 1	0	0	− 1	0	0	2	Low
	CPR	RCTs	1	142	RR 1.30 (0.80–2.10)	− 1	0	0	− 1	0	0	2	Low
Endometrial injury	LBR	RCTs	3	376	RR 1.55 (0.81–2.94)	− 1	0	0	− 1	0	0	2	Low
	CPR	RCTs	3	376	RR 1.43 (0.79–2.61)	− 1	0	0	− 1	0	0	2	Low
	IR	RCTs	1	101	RR 1.70 (1.01–2.84)	− 1	0	0	− 1	0	0	2	Low
	MR	RCTs	3	376	RR 1.39 (0.55–3.53)	− 1	0	0	− 1	0	0	2	Low
	CPR	Observational studies	2	200	OR 3.03 (1.48–6.18)	− 1	0	0	− 1	0	+ 1 (magnitude)	1	Very low
LMWH	LBR	RCTs	1	71	RR 1.38 (0.64–2.96)	− 2	0	0	− 1	0	0	1	Very low
	CPR	RCTs	2	218	RR 1.39 (0.87–2.23)	− 2	0	0	− 1	0	0	1	Very low
	LBR	Observational studies	1	91	OR 1.50 (0.59–3.82)	− 1	0	0	0	0	0	1	Very low
	CPR	Observational studies	1	91	OR 1.42 (0.58–3.45)	− 1	0	0	0	0	0	1	Very low
Hysterosocpy	LBR	RCTs	1	230	RR 0.96 (0.69–1.32)	0	0	0	− 1	0	0	3	Moderate
PGT-A	LBR	RCTs	1	91	RR 1.72 (0.98–3.02)	− 1	0	0	− 1	0	0	2	Low
	CPR	RCTs	1	91	RR 1.86 (1.11–3.12)	− 1	0	0	− 1	0	0	2	Low
	IR	RCTs	1	91	RR 1.71 (0.99–2.94)	− 1	0	0	− 1	0	0	2	Low
	MR	RCTs	1	91	RR 3.58 (0.42–30.83)	− 1	0	0	− 1	0	0	2	Low
	LBR	Observational studies	2	219	OR 0.83 (0.33–2.07)	0	0	0	0	0	0	1	Low
	CPR	Observational studies	3	295	OR 1.58 (0.35–7.12)	− 1	0	0	0	0	0	1	Very low
Atosiban	CPR	Observational studies	1	88	OR 2.63 (1.08–6.40)	− 1	0	0	0	0	0	1	Very low
	IR	Observational studies	1	88	OR 3.12 (1.54–6.28)	− 1	0	0	0	0	0	1	Very low
	MR	Observational studies	1	88	OR 1.66 (0.43–6.35)	− 1	0	0	0	0	0	1	Very low
IVIG	LBR	Observational studies	1	283	OR 1.76 (1.08–2.89)	− 1	0	0	0	0	0	1	Very low
	CPR	Observational studies	1	283	OR 2.08 (1.28–3.36)	− 1	0	0	0	0	0	1	Very low
	IR	Observational studies	1	283	OR 1.43 (1.06–1.94)	− 1	0	0	0	0	0	1	Very low
hCG	LBR	Observational studies	1	67	OR 1.78 (1.02–3.09)	− 1	0	0	0	0	0	1	Very low
	CPR	Observational studies	2	166	OR 1.81 (1.23–2.65)	− 1	0	0	0	0	0	1	Very low
Blastocyst-stage ET	LBR	RCTs	1	54	RR 1.35 (0.30–6.08)	− 1	0	0	− 1	0	0	2	Low
	CPR	RCTs	1	54	RR 1.68 (0.51–5.59)	− 1	0	0	− 1	0	0	2	Low
	IR	RCTs	1	54	RR 3.54 (1.28–9.77)	− 1	0	0	− 1	0	0	2	Low
	MPR	RCTs	1	54	RR 0.90 (0.16–4.95)	− 1	0	0	− 1	0	0	2	Low
ZIFT	LBR	Observational studies	2	314	OR 3.43 (0.03–43.80)	− 1	0	0	0	0	0	1	Very low
	CPR	Observational studies	4	454	OR 2.40 (0.52–11.05)	− 1	0	0	0	0	0	1	Very low
	IR	Observational studies	2		OR 3.73 (0.69–20.27)	− 1	0	0	0	0	0	1	Very low
	MR	Observational studies	1	250	OR 2.09 (0.70–6.21)	− 1	0	0	0	0	0	1	Very low
	MPR	Observational studies	1	250	OR 0.26 (0.07–0.91)	− 1	0	0	0	0	0	1	Very low
PBMC	LBR	RCTs	1	198	RR 2.41 (1.40–4.16)	− 1	0	0	− 1	0	+ 1 (magnitude)	3	Moderate
	CPR	RCTs	3	363	RR 2.18 (1.58–3.00)	− 1	0	0	− 1	0	+ 1 (magnitude)	3	Moderate
	LBR	Observational studies	2	90	OR 3.73 (1.13–12.29)	− 1	0	0	− 1	0	+ 1 (magnitude)	1	Very low
	CPR	Observational studies	3	306	OR 2.03 (1.22–3.36)	− 1	0	0	− 1	0	+ 1 (magnitude)	1	Very low
	IR	Observational studies	2	90	OR 4.54 (1.82–11.35)	− 1	0	0	− 1	0	+ 1 (magnitude)	1	Very low
AH	CPR	RCTs	1	207	RR 0.78 (0.48–1.27)	− 1	0	0	− 1	0	0	2	Low
	LBR	Observational studies	1	109	OR 0.52 (0.13–2.09)	− 1	0	0	0	0	0	1	Very low
	CPR	Observational studies	1	109	OR 1.42 (0.45–4.48)	− 1	0	0	0	0	0	1	Very low
	MPR	Observational studies	1	109	OR, 1.49 (0.09–24.44)	− 1	0	0	0	0	0	1	Very low
PRP	CPR	RCTs	2	195	RR 2.45 (1.55–3.86)	− 1	0	0	− 1	0	0	2	Low

GRADE Working Group grades of evidence. High quality: we are very confident that the true effect lies close to that of the estimate of the effect. Moderate quality: we are moderately confident in the effect estimate: the true effect is likely to be close to the estimate of the effect, but there is a possibility that it is substantially different. Low quality: our confidence in the effect estimate is limited: the true effect may be substantially different from the estimate of the effect. Very low quality: we have very little confidence in the effect estimate: the true effect is likely to be substantially different from the estimate of effect.

*RCT* randomized clinical trial, *G-CSF* granulocyte-colony stimulating factor, *LMWH* low molecular weight heparin, *PBMC* peripheral blood mononuclear cells, *AH* assisted hatching, *PGT-A* preimplantation genetic testing for aneuploidy, *IVIG* intravenous immunoglobulin, *PRP* platelet rich plasma, *hCG* human chorionic gonadotropin, *ZIFT* zygote intrafallopian transfer, *IR* implantation rate, *CPR* clinical pregnancy rate, *MR* miscarriage rate, *MPR* multiple pregnancy rate, *LBR* live birth rate, *95% CI* 95% confidence interval, *RR* risk ratio, *OR* odds ratio.

### Synthesis of results

#### Uterine interventions

##### Intentional Endometrial injury

Three RCTs^8,146,164^ and two observational studies^152,159^ evaluated the impact of an intentional injury to the endometrium during the spontaneous menstrual cycles before IVF on the outcomes of the IVF cycle.

*Primary outcomes* Meta-analysis of RCTs did not show significantly increased chances of pregnancy and live birth in women who underwent intentional endometrial injury (random effects model, RR 1.43; 95% CI 0.79–2.61; p = 0.24; I^2^ = 52% and random effects model, 1.55; 95% CI 0.81–2.94; p = 0.18; I^2^ = 46%, respectively)^8,146,164^ (Fig. 4). On the contrary, pooling of results from observational studies showed a beneficial effect of endometrial injury on pregnancy rate (fixed effects model, OR 3.03; 95% CI 1.48–6.18; p = 0.002; I^2^ = 0%)^152,159^ (Fig. 4).

*Secondary outcomes* Steengaard Olesen et al. observed a slight benefit of endometrial injury on implantation rate (RR 1.70; 95% CI 1.01–2.84; p = 0.04)^164^. Meta-analysis of RCTs did not show any impact on MR (fixed effects model, RR 1.39; 95% CI 0.55–3.53; p = 0.48; I^2^ = 0%)^8,146,164^.

*Subgroup analysis* Gurgan et al., performed endometrial injury on the 10th–12th day of the late follicular phase; Baum et al., on days 9–12 and 21–24 of the menstrual cycle and Steengaard Olesen et al. at menstrual cycle day 18–22^8,146,164^.

Analyzing the results of the studies separately, no benefits were observed for the endometrial injury performed solely in the follicular phase (CPR, RR 1.65; 95% CI 0.98–2.77; p = 0.06 and LBR, RR 1.79; 95% CI 0.99–3.24; p = 0.05)^146^. Steengaard Olesen et al. observed an increased chance of clinical pregnancy (RR 1.72; 95% CI 1.05–2.83; p = 0.03) in treated subjects but failed to confirm this positive impact on LBR (RR 1.74; 95% CI 0.99–3.05; p = 0.05)^164^. Baum et al. did not observe a significant effect on both outcomes (CPR, RR 0.20; 95% CI 0.03–1.55; p = 0.12 and LBR, RR 0.11; 95% CI 0.01–1.92; p = 0.13)^8^. Gurgan et al. were also the only ones who performed the endometrial injury via hysteroscopy^146^.

*Quality of the evidence* We downgraded the quality of the evidence provided by RCTs by one level for risk of bias and, considering the low number of events, by one level for imprecision. The quality of the evidence provided by observational studies was downgraded by one level for risk of bias and, considering the wide confidence interval, by one level for imprecision and upgraded by one level for the large magnitude of the effect (Table 4).

##### Hysteroscopy

One RCT investigated whether outpatient hysteroscopy in the month before starting IVF treatment cycle could improve the outcome in women with RIF^143^.

*Primary outcomes* 144 failed to show an increase in live birth chances (RR 0.96; 95% CI 0.69–1.32; p = 0.79)^143^ (Fig. 4).

*Quality of the evidence* The data reported in the present meta-analysis were extrapolated from a sub-analysis carried out by El-Thouky et al.^143^. Furthermore, the number of events is low. Hence, we downgraded the quality of the evidence by one level for imprecision (Table 4).

##### Atosiban

One observational study^12^ examined the effect of atosiban administered before transfer of frozen-thawed embryo to women with RIF.

*Primary outcomes* Authors observed an increased CPR in treated women when compared to controls (OR 2.63; 95% CI 1.08–6.40; p = 0.03)^12^ (Fig. 4).

*Secondary outcomes* 148 showed an effect on chances of embryo implantation (OR 3.12; 95% CI 1.54–6.28; p = 0.002) and did not find any impact of miscarriage risk (OR 1.66; 95% CI 0.43–6.35; p = 0.46) of atosiban administration^12^.

*Quality of the evidence* The quality of the evidence provided by He et al. was downgraded by one level for risk of bias (Table 4).

#### Laboratory and procedural technologies and interventions

##### Sequential ET

One RCT^13^ and two observational studies^75,144^ compared sequential ET (cleavage stage ET followed by blastocyst ET) vs blastocyst stage ET in women with RIF.

*Primary outcomes* Meta-analysis of observational studies showed an increased chance of clinical pregnancy in women who underwent sequential ET (fixed effects model, OR 2.64; 95% CI 1.56–4.47; p = 0.0003; I^2^ = 0%)^75,144^ (Fig. 4). On the contrary, Shahrokh Tehraninejad et al. failed to show a beneficial effect (RR 1.04; 95% CI 0.67–1.63; p = 0.85)^13^ (Fig. 4).

*Secondary outcomes* Fang et al., observed a beneficial effect of sequential ET on implantation rate (OR 2.95; 95% CI 1.65–5.27; p = 0.0003) (Fang et al., 2013). Meta-analysis of observational studies^75,144^ and Shahrokh Tehraninejad et al. did not show an impact on MPR (fixed effects model, OR 2.38; 95% CI 0.87–6.47; p = 0.09; I^2^ = 36% and RR 1.13; 95% CI 0.47–2.72; p = 0.79, respectively).

*Quality of the evidence* We downgraded the quality of the evidence provided by Shahrokh Tehraninejad et al. by one level for risk of bias and, considering the low number of events, by one level for imprecision. The quality of the evidence provided by observational studies was downgraded by one level for risk of bias (Table 4).

##### PGT-A

Two RCTs^20,141^ and three observational studies^145,160,167^ investigated the potential role of PGT-A in improving IVF outcomes in women with RIF.

*Primary outcomes* Meta-analysis of RCTs failed to show an improvement in both clinical pregnancy and live birth chances (random effects model, RR 1.07; 95% CI 0.36–3.15; p = 0.90; I^2^ = 89% and RR 0.98; 95% CI 0.32–2.94; p = 0.97; I^2^ = 87%) in women who underwent PGT-A^20,141^ (Fig. 4).

Pooling of results of observational studies did not show a beneficial effect of PGT-A on both pregnancy (random effects model, OR 1.58; 95% CI 0.35–7.12; p = 0.55; I^2^ = 86%)^145,160,167^ and live birth chances (random effects model, OR 0.83; 95% CI 0.33–2.07; p = 0.69; I^2^ = 44%)^160,167^ (Fig. 4).

*Secondary outcomes* Rubio et al. did not observe an impact of PGT-A on chances of embryo implantation and miscarriage in women who underwent PGT-A (RR 1.71; 95% CI 0.99–2.94; p = 0.05 and RR 3.58; 95% CI 0.42–30.83; p = 0.25, respectively)^20^.

*Quality of the evidence* The evidence emerged from RCTs was downgraded by one level for risk of bias and, considering the low number of events, by one level for imprecision. For CPR, we downgraded the quality of the evidence provided by observational studies by one level for risk of bias. For LBR, we did not downgrade the quality of the evidence (Table 4).

##### Blastocyst-stage ET

One RCT compared blastocyst-stage ET outcomes with day 2–3 ET outcomes in women who failed to conceive after three or more day 2–3 IVF/ET cycles^149^.

*Primary outcomes* Levitas et al. failed to show a benefit of this strategy on both CPR (RR 1.68; 95% CI 0.51–5.59; p = 0.39) and LBR (RR 1.35; 95% CI 0.30–6.08; p = 0.70)^149^.

*Secondary outcomes* Authors observed a significantly increased chance of embryo implantation in treated women (RR 3.54; 95% CI 1.28–9.77; p = 0.01)^149^. MPR did not result significantly different between groups (RR 0.90; 95% CI 0.16–4.95; p = 0.90)^149^.

*Quality of the evidence* The quality of the evidence was downgraded by one level for risk of bias and, considering the low number of events, by one level for imprecision (Table 4).

##### ZIFT

Three observational studies investigated the possible beneficial effect of ZIFT in women with RIF^18,150,163^.

*Primary outcomes* Meta-analysis did not show increased chances of clinical pregnancy (random effects model, OR 2.40; 95% CI 0.52–11.05; p = 0.26; I^2^ = 87%)^18,150,163^ and live birth (random effects model, OR 3.43; 95% CI 0.03–43.80; p = 0.62; I^2^ = 91%) in women who underwent ZIFT (Fig. 4).

*Secondary outcomes* Pooling of results failed to show a benefit on embryo implantation chances (random effects model, OR 3.73; 95% CI 0.69–20.27; p = 0.13; I^2^ = 64%)^18,150^. MPR resulted significantly lower in women who underwent ZIFT (OR 0.26; 95% CI 0.07–0.91; p = 0.04)^163^. Shahrokh Tehraninejad et al. did not observe an impact on MR (OR 2.09; 95% CI 0.70–6.21; p = 0.19)^163^.

*Quality of the evidence* The quality of the evidence was downgraded by one level for risk of bias (Table 4).

##### AH

One RCT^153^ and one observational study^158^ investigated the effect of AH on IVF outcomes in women with RIF.

*Primary outcomes* 156 did not observe an increased chance of clinical pregnancy in women who underwent AH (RR 0.78; 95% CI 0.48–1.27; p = 0.31)^153^ (Fig. 4).

Primi et al., confirmed this finding (CPR, OR 1.42; 95% CI 0.45–4.48; p = 0.55) and failed to show a beneficial effect also on chances of live birth (OR 1.92; 95% CI 0.48–7.67; p = 0.36)^158^ (Fig. 4).

*Secondary outcomes* Primi et al. did not observed any difference in MPR between groups (OR, 1.49; 95% CI 0.09–24.44; p = 0.78)^158^*.*

*Quality of the evidence* The quality of the evidence provided by Rufas-Sapir et al. was downgraded by one level for risk of bias and, considering the low number of events, by one level for imprecision^2^. We downgraded the quality of the evidence emerged from the study conducted by Primi et al.*,* by one level for risk of bias (Table 4).

#### Immunomodulatory therapies

##### G-CSF administration

Six RCTs evaluated the possible beneficial effect of the subcutaneous or intrauterine G-CSF administration^140,142,147,148,161,162^.

*Primary outcomes* Pooling of results from studies showed increased chances pregnancy in treated subjects (fixed effects model, 1.94; 95% CI 1.47–2.55; p < 0.00001; I^2^ = 0%)^140,142,147,148,161,162^. Only one study investigated the impact of intrauterine G-CSF infusion on the chances of live birth and failed to show a benefit (RR 0.84; 95% CI 0.41–1.73; p = 0.64)^147^.

*Secondary outcomes* Two trials reported implantation rate. Pooling of results showed a beneficial effect (fixed effects model, RR 2.41; 95% CI 1.38–4.22; p = 0.002; I^2^ = 0%)^140,142^. Kalem et al. did not observe any impact on MR (RR 3.20; 95% CI 0.69–14.93; p = 0.14)^147^.

**Figure 3:**
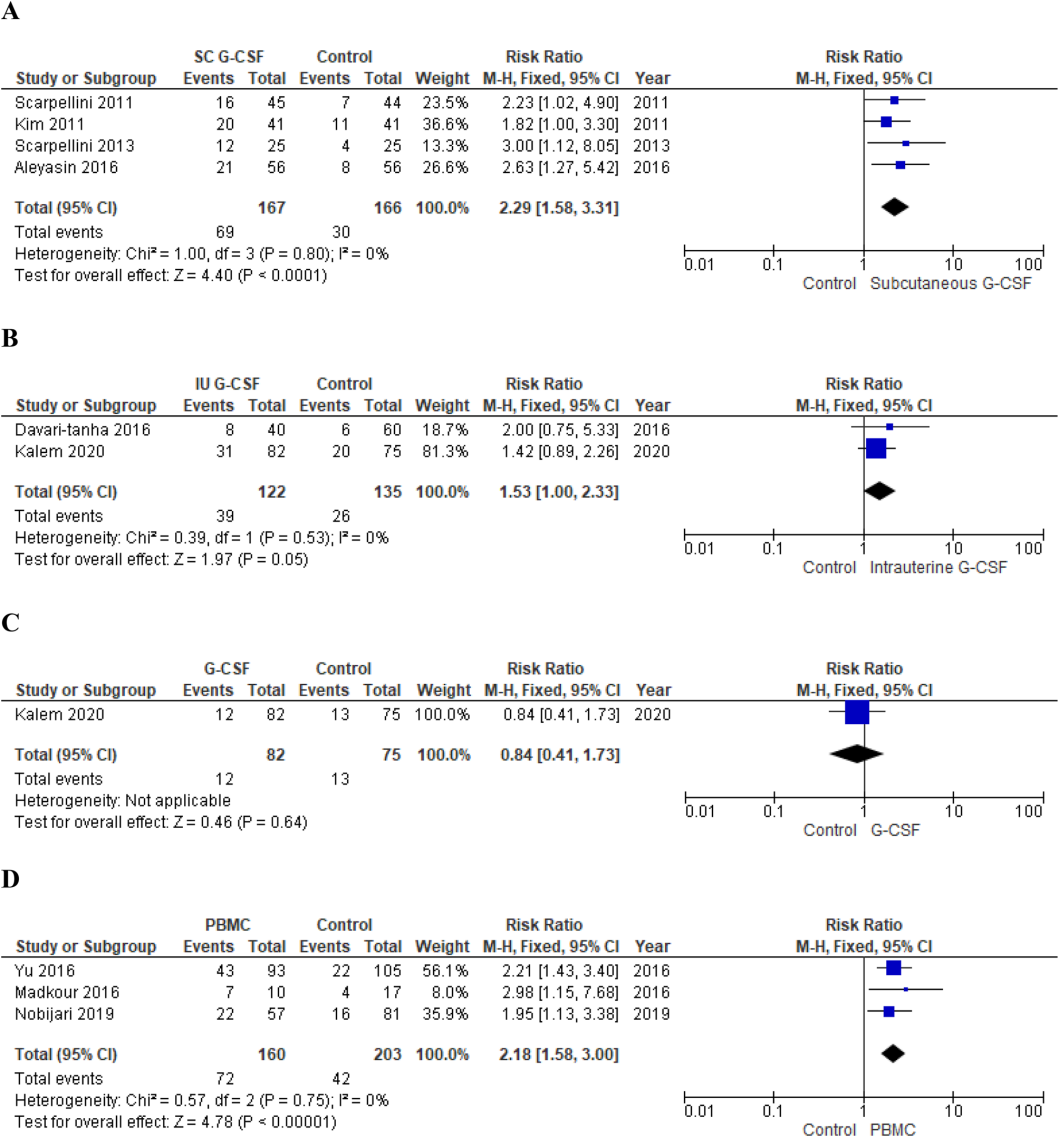

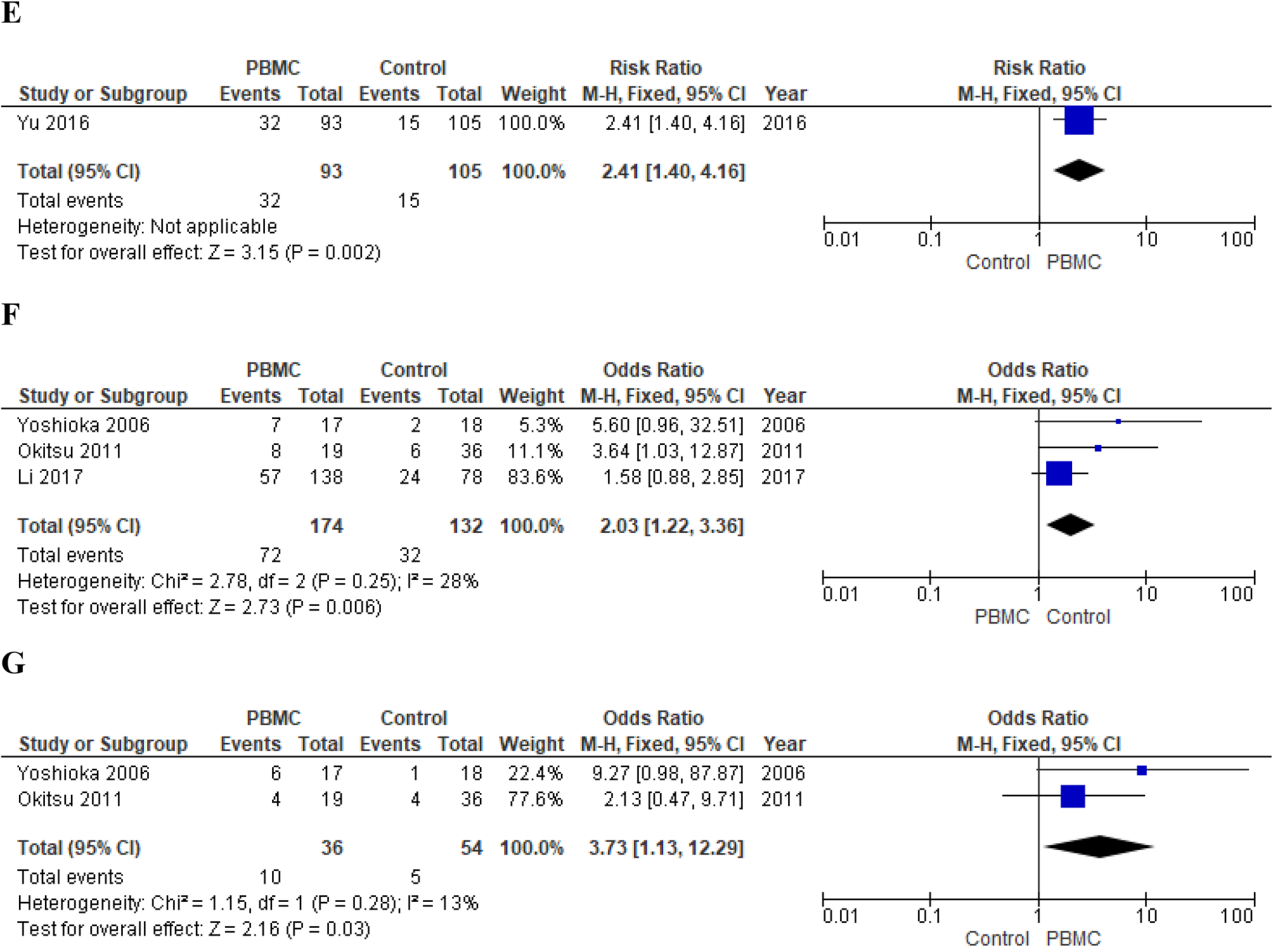
(**A**) Effect of subcutaneous G-CSF administration on CPR in women with RIF (RCTs). (**B**) Effect of intrauterine G-CSF infusion on CPR in women with RIF (RCTs). (**C**) Effect of subcutaneous G-CSF administration on LBR in women with RIF (RCT). (**D**) Effect of intrauterine PBMC infusion on CPR in women with RIF (RCTs). (**E**) Effect of intrauterine PBMC infusion on LBR in women with RIF (RCT). (**F**) Effect of intrauterine PBMC infusion on CPR in women with RIF (observational studies). (**G**) Effect of intrauterine PBMC infusion on LBR in women with RIF (observational studies). *RIF* repeated implantation failure, *G-CSF* granulocyte-colony stimulating factor, *PBMC* peripheral blood mononuclear cells, *RCT* randomized clinical trial, *CPR* clinical pregnancy rate, *LBR* live birth rate.

*Subgroup analysis* Subcutaneous and intrauterine route of administration were analyzed separately (Fig. 3). Subcutaneous G-CSF administration resulted associated with an increased chance of clinical pregnancy (fixed effects model, RR 2.29; 95% CI 1.58–3.31; p < 0.0001; I^2^ = 0%) when compared with no treatment^140,148,161,162^ (Fig. 3). On the contrary, intrauterine administration had no impact on CPR (fixed effects model, RR 1.53; 95% CI 1.00–2.33; p = 0.05; I^2^ = 0%)^142,147^ (Fig. 3). Aleyasin et al. who investigated the subcutaneous route of administration observed a positive effect on embryo implantation chances (RR 2.94; 95% CI 1.24–5.01; p = 0.01)^140^. In contrast, Davari-tanha et al. who focused on intrauterine G-CSF injection did not observe any impact on IR (RR 2.28; 95% CI 0.90–5.74; p = 0.08)^142^.

*Quality of the evidence* In the majority of RCTs, the description of allocation concealment was unclear or the treatment providers were not blinded, hence we downgraded the quality of the evidence by one level for risk of bias for all outcomes. Considering the low total number of events, we also downgraded the quality of the evidence by one level for imprecision for all outcomes. For CPR evaluated in studies focused on subcutaneous G-CSF administration, we upgraded the quality of evidence by one level for the large magnitude of the effect (Table 4).

##### Intravenous intralipid infusion

One RCT investigated the effect of the intravenous infusion of intralipid^27^.

**Figure 4:**
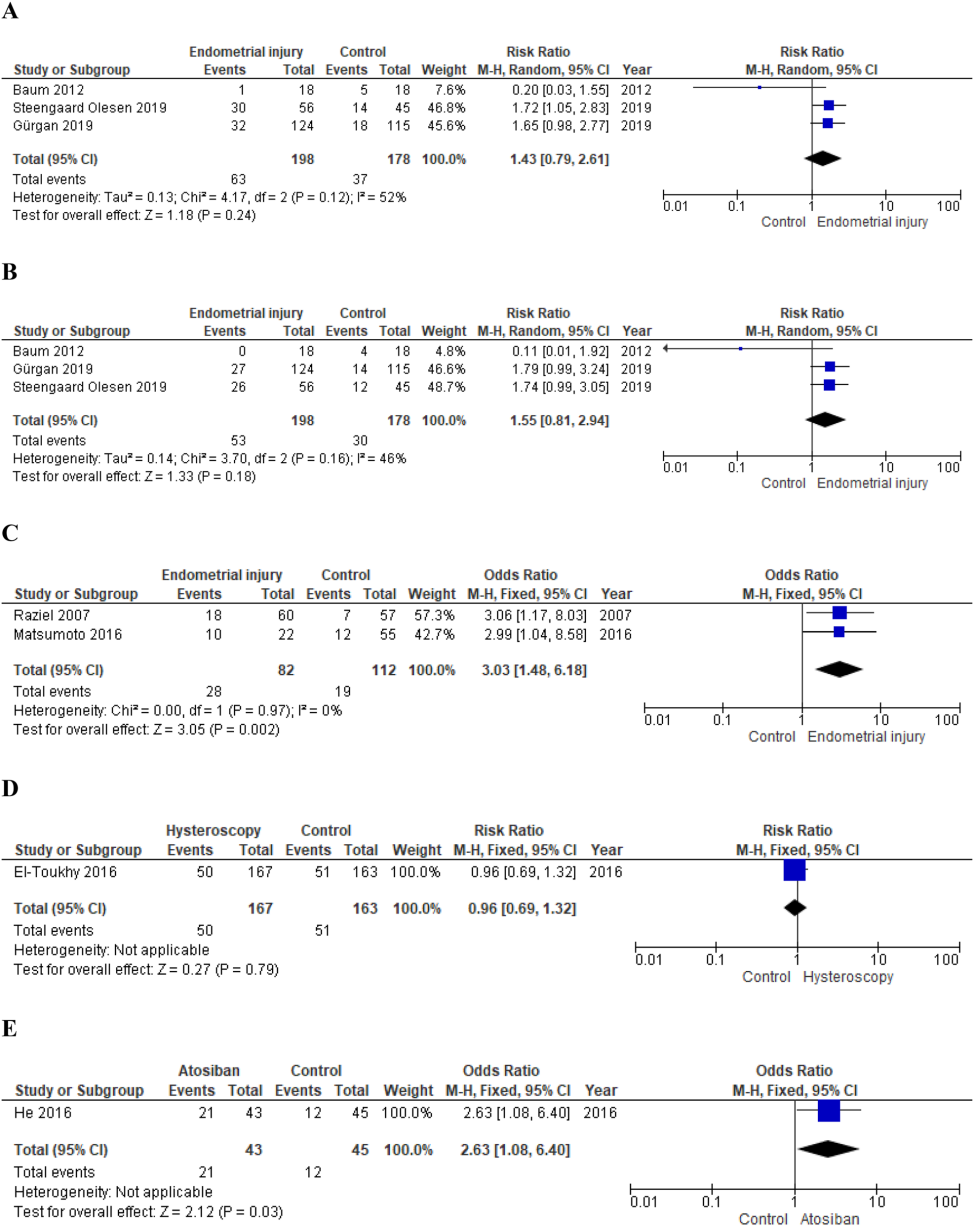

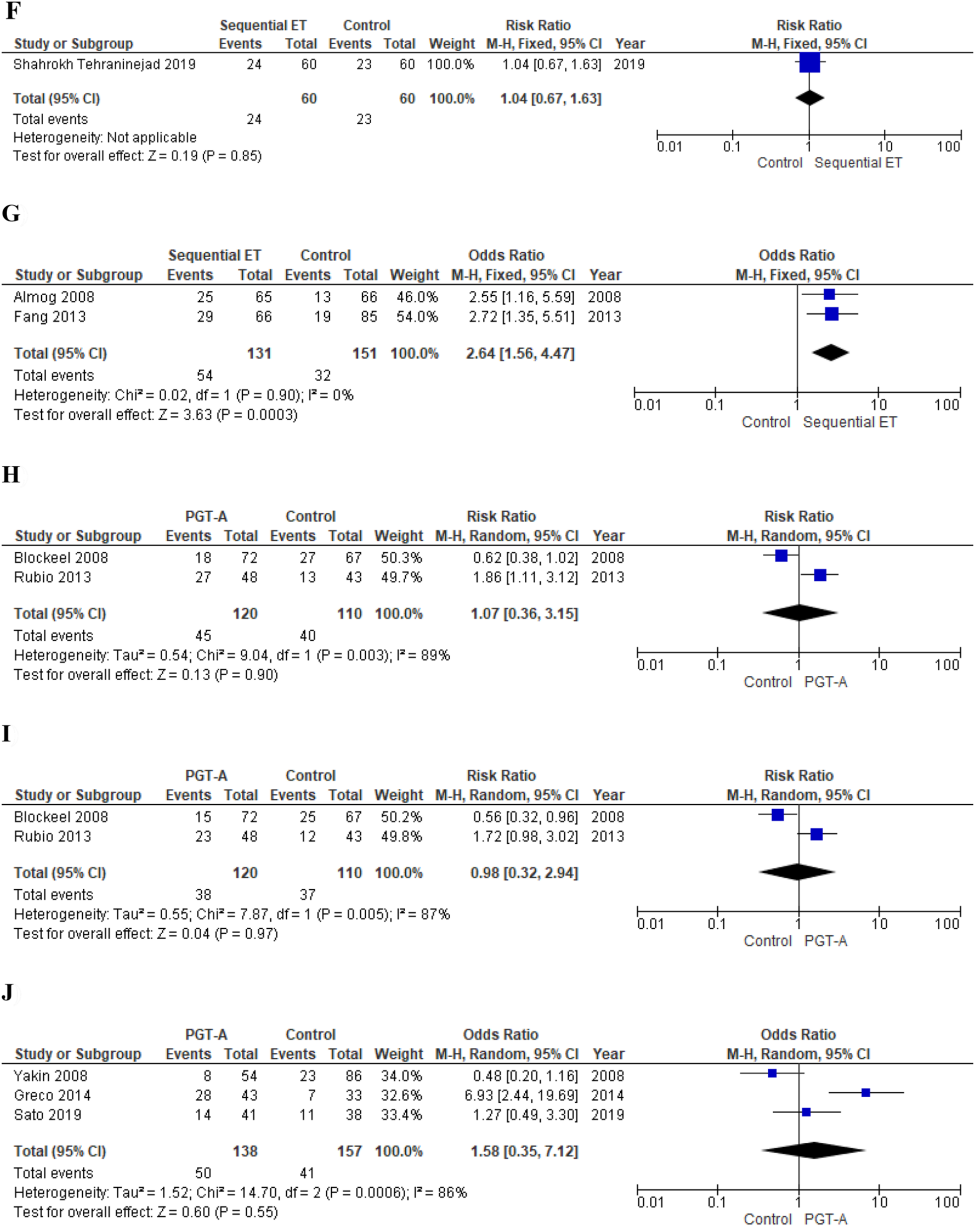

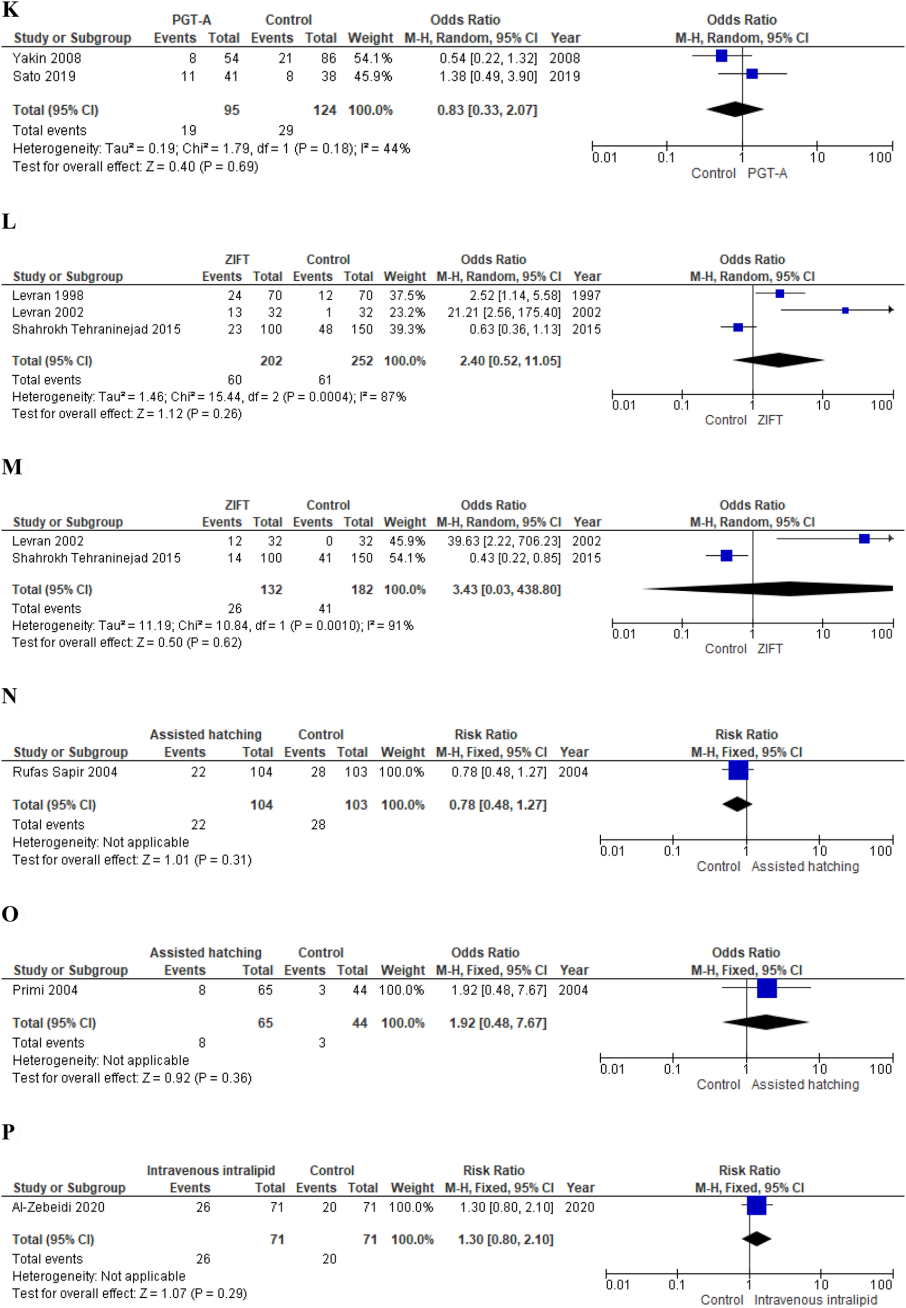

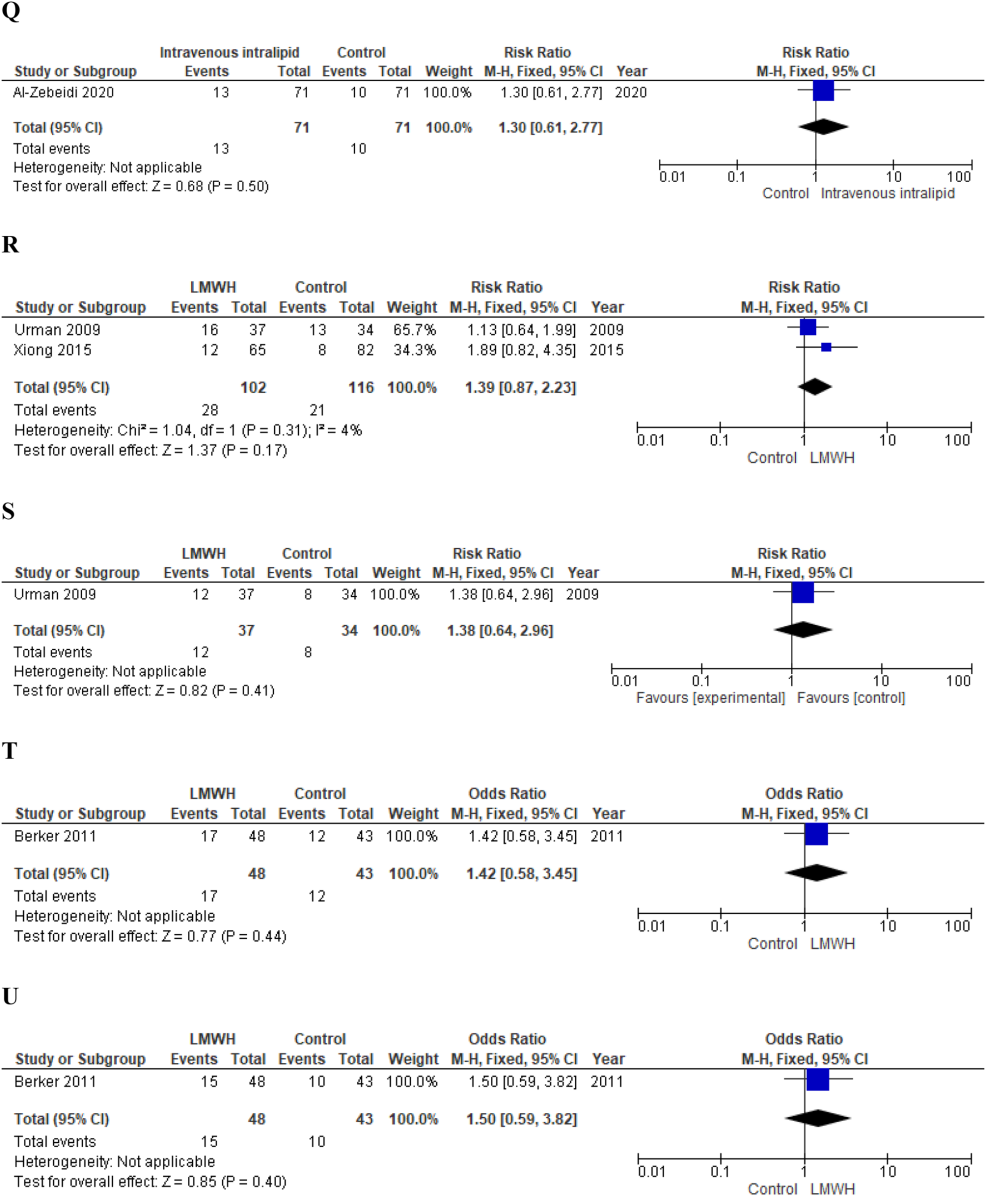

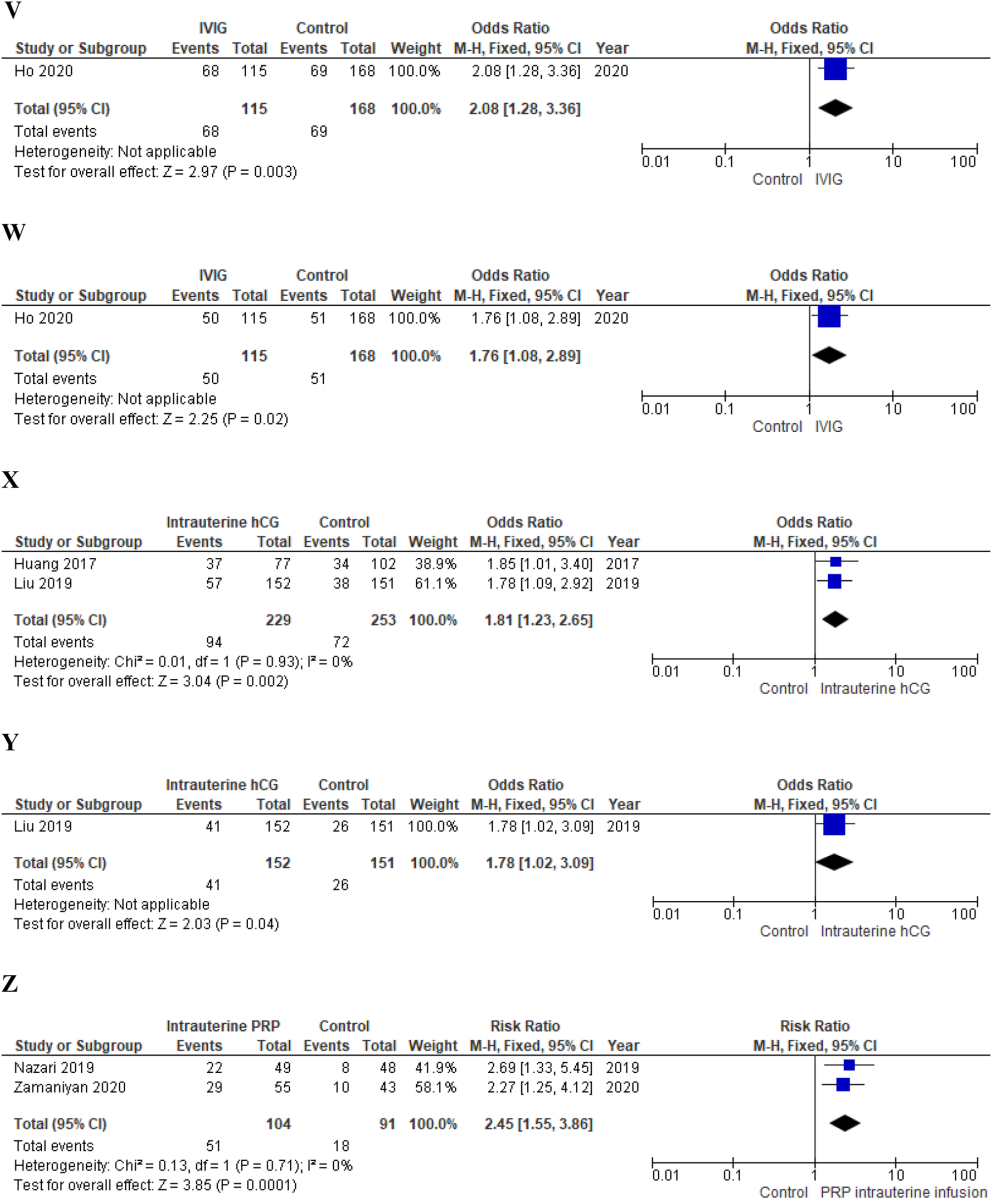
(**A**) Effect of intentional endometrial injury on CPR in women with RIF (RCTs). (**B**) Effect of intentional endometrial injury on LBR in women with RIF (RCTs). (**C**) Effect of intentional endometrial injury on CPR in women with RIF (observational studies). (**D**) Effect of hysteroscopy on LBR in women with RIF (RCT). (**E**) Effect of atosiban on CPR in women with RIF (observational study). (**F**) Effect of sequential ET on CPR in women with RIF (RCT). (**G**) Effect of sequential ET on CPR in women with RIF (observational studies). (**H**) Effect of PGT-A on CPR in women with RIF (RCTs). (**I**) Effect of PGT-A on LBR in women with RIF (RCTs). (**J**) Effect of PGT-A on CPR in women with RIF (observational studies). (**K**) Effect of PGT-A on LBR in women with RIF (observational studies). (**L**) Effect of ZIFT on CPR in women with RIF (observational studies). (**M**) Effect of ZIFT on LBR in women with RIF (observational studies). (**N**) Effect of AH on CPR in women with RIF (RCT). (**O**) Effect of AH on LBR in women with RIF (observational study). (**P**) Effect of intravenous intralipid on CPR (RCT). (**Q**) Effect of intravenous intralipid on LBR in women with RIF (RCT). (**R**) Effect of LMWH on CPR in women with RIF (RCTs). (**S**) Effect of LMWH on LBR in women with RIF (RCT). (**T**) Effect of LMWH on CPR in women with RIF (observational study). (**U**) Effect of LMWH on LBR in women with RIF (observational study). (**V**) Effect of IVIG on CPR in women with RIF (observational study). (**W**) Effect of IVIG on LBR in women with RIF (observational study). (**X**) Effect of intrauterine hCG infusion on CPR in women with RIF (observational studies). (**Y**) Effect of intrauterine hCG infusion on LBR in women with RIF (observational study). (**Z**) Effect of intrauterine PRP infusion on CPR in women with RIF (RCT). *ET* embryo transfer, *RIF* repeated implantation failure, *RCT* randomized clinical trial, *CPR* clinical pregnancy rate, *LBR* live birth rate, *LMWH* low molecular weight heparin, *PGT-A* preimplantation genetic testing for aneuploidy, *IVIG* intravenous immunoglobulin, *hCG* human chorionic gonadotropin, *ZIFT* zygote intrafallopian transfer, *AH* assisted hatching, *PRP* platelet rich plasma.

*Primary outcomes* Authors failed to show a benefit of the intravenous intralipid infusion on both the clinical pregnancy rate and the live birth rate (RR 1.30; 95% CI 0.80–2.10; p = 0.29 and 1.30; 95% CI 0.61–2.77, respectively) (Fig. 4).

*Quality of the evidence* Quality of the evidence was downgraded by one level for risk of bias and by one level for imprecision (Table 4).

##### LMWH

Two RCTs^165,166^ and one observational study^31^ investigated the effect of subcutaneous LMWH administration.

*Primary outcomes* Meta-analysis of RCTs failed to show a beneficial effect on both CPR (RR 1.39; 95% CI 0.87–2.23; p = 0.17; I^2^ = 4%)^165,166^ and LBR (RR 1.38; 95% CI 0.64–2.96; p = 0.41)^165^. Berker et al. also did not observe a significant increase of pregnancy and live birth chances (OR 1.42, 95% CI 0.58–3.45; p = 0.44 and OR 1.50; 95% CI 0.59–3.82; p = 0.40, respectively) (Fig. 4).

*Quality of the evidence* The quality of the evidence provided by RCTs was downgraded by two levels for risk of bias and by one level for imprecision. We also downgraded the level of the evidence provided by Berker et al. by one level for risk of bias (Table 4).

##### IVIG

One observational study^21^ evaluated the efficacy of IVIG in women with RIF.

*Primary outcomes* Chances of clinical pregnancy and live birth resulted significantly increased in treated women (OR 2.08; 95% CI 1.28–3.36; p = 0.003 and OR 1.76; 95% CI 1.08–2.89; p = 0.02, respectively)^21^ (Fig. 4).

*Secondary outcomes* Ho et al., observed an increased chance of embryo implantation (OR 1.43; 95% CI 1.06–1.94; p = 0.02) in treated subjects^21^.

*Quality of the evidence* The quality of the evidence was downgraded by one level for risk of bias (Table 4).

##### Intrauterine hCG injection

Two observational studies investigated the effect of intrauterine hCG injection in women with RIF^28,151^.

*Primary outcomes* Chances of clinical pregnancy (fixed effects model, OR 1.81; 95% CI 1.23–2.65; p = 0.002; I^2^ = 0%)^28,151^ and live birth (OR 1.78; 95% CI 1.02–3.09; p = 0.04)^151^ resulted significantly increased in treated women (Fig. 4).

*Secondary ooutcomes* Liu et al. showed a beneficial effect of intrauterine hCG injection on implantation rate (OR 1.71; 95% CI 1.08–2.71; p = 0.02)^151^.

*Quality of the evidence* The quality of the evidence was downgraded by one level for risk of bias (Table 4).

##### Intrauterine PBMC infusion

Three RCTs^154,156,169^ and three observational studies^24,157,168^ investigated the effect of intrauterine administration of autologous PBMC on IVF outcomes in women with RIF.

*Primary outcomes* Meta-analysis of RCTs showed a significant increase in chances of clinical pregnancy (fixed effects model, RR 2.18; 95% CI 1.58–3.00; p < 0.00001; I^2^ = 0%)^154,156,169^ and live birth (RR 2.41; 95% CI 1.40–4.16; p = 0.002)^169^ in treated women (Fig. 3). Pooling of results of observational studies confirmed the positive effect on both CPR (fixed effects model, OR 2.03; 95% CI 1.22–3.36; p = 0.006; I^2^ = 28%)^24,157,168^ and LBR (fixed effects model, OR 3.73; 95% CI 1.13–12.29; p = 0.03; I^2^ = 13%)^157,168^ (Fig. 3).

*Secondary outcomes* Meta-analysis of observational studies showed an increased chance of embryo implantation in treated women (fixed effects model, OR 4.54; 95% CI 1.82–11.35; p = 0.001; I^2^ = 0%)^157,168^.

*Quality of the evidence* The quality of the evidence provided by RCTs was downgraded by one level for risk of bias, by one level for imprecision and upgraded by one level for the large magnitude of the effect (Table 4). The quality of the evidence provided by observational studies was downgraded by one level for risk of bias and by one level for imprecision and upgraded by one level for the large magnitude of the effect (Table 4).

##### Intrauterine PRP infusion

Two RCTs^155,170^ investigated whether administration of intrauterine PRP could improve IVF outcomes in women with RIF.

*Primary outcomes* Pooling of results showed a significantly increased chance of clinical pregnancy in treated women (fixed effects model, RR 2.45; 95% CI 1.55–3.86; p = 0.0001; I^2^ = 0%)^155,170^ (Fig. 4)*.*

*Quality of the evidence* The quality of the evidence was downgraded by one level for risk of bias and, considering the low number of events, by one level for imprecision (Table 4).

## Discussion

In the present study, meta-analysis of RCTs showed a beneficial effect of PBMC intrauterine infusion on both LBR and CPR and of subcutaneous G-CSF administration and intrauterine PRP infusion on CPR in women with RIF. Pooling of results of observational studies also demonstrated a positive effect of IVIG and hCG intrauterine infusion on both CPR and LBR and of atosiban administration on CPR. Meta-analysis of studies investigating the possible impact of intrauterine G-CSF infusion, LMWH, hysteroscopy, blastocyst-stage ET, ZIFT, PGT-A and AH failed to observe an impact on IVF outcome. Results about the effects of sequential ET and intentional endometrial injury are conflicting. The quality of the evidence that emerged from RCTs investigating the effect of intrauterine PBMC infusion and subcutaneous G-CSF administration was moderate. For all other therapies/interventions it varied from low to very low.

Among the therapies that have been proven to be potentially effective, the intrauterine infusion of PBMC is supported by the most convincing evidence. In fact, meta-analyses of RCTs and of observational studies agree in demonstrating the positive effect on both primary outcomes and the magnitude of calculated effect estimates is considerable. Pourmoghadam et al. in an interesting meta-analysis had already shown a beneficial effect in women with at least three IVF failures^171^. The subsequent publication of the study conducted by Nobijari et al.^156^, which was the first RCT to report the chances of live birth, further strengthened the evidence. Nevertheless, data on the impact on the LBR as well as on the safety profile of this therapy should still be considered scanty.

The administration of G-CSF also emerged as a promising treatment option in women with RIF. Our findings confirmed those recently published by Kamath et al. who showed that in women with two or more IVF failures, G-CSF administration may improve CPR versus placebo^47^. Interestingly, we observed that of the two possible routes of administration, the only potentially effective seems to be the systemic one. Importantly, the magnitude of the effect was considerable and, as a consequence, we upgraded the quality of the evidence to moderate. Unfortunately, no data about the rate of live birth can be extracted from included studies that investigated this route of administration, which may impair the convincingness of the analysis. Reasons for discrepancies between the effects of systemic and intrauterine administration have yet to be fully elucidated. One could speculate that when administered systemically, G-CSF has a positive effect on oocyte maturation and embryonic development, while in locally endometrial cavity applications oocytes and embryos are deprived of this positive support^147^.

Intrauterine hCG infusion constitutes an excellent candidate to be tested in women with RIF. In fact, by acting as the homologous isomer of LH, hCG shares a common receptor with LH, namely, LHCGR, and their combination can regulate both endometrium receptivity and embryo implantation^172^. Importantly, in a recent meta-analysis, Gao et al., showed that infertile women who received intrauterine hCG injection before ET exhibited significantly higher rates of implantation, ongoing pregnancy and live birth and a lower rate of miscarriage^172^. In the present meta-analysis, pooling of results of observational studies focusing on patients with RIF showed a beneficial effect on both CPR and LBR. Unfortunately, the quality of the evidence was very low. In particular, the different volumes of culture medium (1 ml and 0.2 ml) and doses of hCG (1000 UI and 500 UI) impair the clinical homogeneity between studies and significantly limit the reliability of our results^28,151^.

Hypothesizing a key role of the immune response in the pathogenesis of RIF, IVIG, intravenous intralipid injection and PRP intrauterine infusion have also been proposed as possible treatments. Initial results regarding the efficacy of IVIG and PRP intrauterine injection are encouraging. However, even in these cases, the very low quality of the evidence does not allow reliable conclusions.

The decrease of the frequency and amplitude of uterine contractions obtained through the administration of atosiban, has also been theorized as a method to enhance the probability of embryo implantation and pregnancy in women with RIF. Our results were obtained from the data extrapolated from a single observational study and are in line with those of a recent meta-analysis conducted by Huang et al., who, using less stringent inclusion criteria [i.e. two or more consecutive failed IVF-ET attempts in which at least 1 ± 2 high quality embryos were transferred in each cycle], demonstrated increased chances of implantation, clinical pregnancy and live birth in women with RIF treated with atosiban^28^. Well conducted RCTs focusing on women with RIF diagnosed according to the criteria proposed in the present study are warranted.

Inconclusive results and demonstrations of inefficacy that emerged from the present meta-analysis are of particular importance. Over the years, we witnessed the emergence of a number of RIF treatment options of simple execution but characterized by weak rational bases. Nonetheless, their introduction into current clinical practice occurred rapidly without waiting for adequate evidence of efficacy and safety. Such conduct evidently conflicts with the principle of the traditional medical ethics summarized in the injunction “*primum non nocere*” and with the duty to protect patients, already psychologically frustrated, from false hopes and to avoid waste of resources.

In this perspective, the results about the effect of intentional endometrial injury deserve to be commented. The biological plausibility and relative ease of execution of this intervention attracted the attention of many clinicians around the world. Endometrial scratching is a safe procedure. However, it is somewhat painful. When performed in the luteal phase, patients reported pain scores between 3 and 7 of 10, and the procedure was discontinued due to pain in a number of cases^173^. Its efficacy in women with RIF is debated. Nonetheless, an online survey distributed to 189 fertility clinics across Australia, New Zealand and the UK found that 92% of clinicians recommend endometrial scratching to women with RIF^173^. In our study, meta-analysis of RCTs demonstrated the inefficacy of this intervention in increasing CPR and LBR. On the contrary, pooling of results of observational studies suggested a beneficial effect on CPR. These discrepancies combined with the relatively small sample size of the included studies and the statistical moderate/substantial heterogeneity do not allow conclusive interpretations.

A recent RCT showed a potentially harmful effect of the endometrial biopsy performed in the follicular phase. In fact, authors reported a higher incidence of clinical miscarriages in the context of in-cycle scratching, which led to the study premature halt^174^. This considered, we conducted a sub-analysis on the basis of endometrial injury timing without however observing the superiority of one strategy over the others. Importantly, a recent retrospective study questioned the existence of RIF due to endometrial effect. In a cohort of 4229 women whose endometrium was sonographically normal and who underwent up to three frozen euploid single embryo-transfers, authors found a cumulative sustained implantation rate of 95.2%. As a result, RIF incidence was estimated < 5%^175^.

At present, there is no evidence to support the routine use of hysteroscopy as a screening and treatment tool in the population of women with RIF and a normal uterine cavity on ultrasound or hysterosalpingogram to improve the reproductive success rate. However, available data are scanty. Notably, there is compelling rationale that hysteroscopy might be effective in women with RIF. In fact, intrauterine pathology has been reported in as many as 50% of women with RIF leading to suggest that the correction of such pathology could improve IVF outcome^143^. Benefit could also be due to the negotiation of the cervical canal, thus, facilitating the subsequent embryo transfer^176^. Hysteroscopy has also the considerable advantage of allowing targeted endometrial biopsies. In this regard, a recent interesting meta-analysis showed that chronic endometritis therapy might be beneficial in patients suffering from RIF even if, according to the authors, the body of evidence on this topic is still insufficient to recommend routine chronic endometritis screening as intervention in such patients^37^. Future RCTs are thus welcomed in order to test such multiple hypothetical beneficial function of hysteroscopy in women with RIF.

Notably, we also failed to show a significant impact of LMWH administration on both CPR and LBR in non-thrombophilic women with RIF. However, the reliability of the results is limited by the very low quality of the evidence. Furthermore, the absence of data regarding the undesirable effects of LMWH administration [e.g. risk of bleeding] does not allow to grasp the whole picture.

Pooling of results of studies investigating the possible role of PGT-A did not show a positive effect on both clinical pregnancy and live birth chances per patient. Future research efforts should probably test this intervention on a population of older women in whom one may suspect with higher confidence that aneuploidy constitutes the cause of RIF. In this regard, it has however to be highlighted that PGT-A cannot be expected to increase the chance of live birth per patient^177^. It can at most only alleviate the burden of treatment to patients by reducing the number of transfers.

Finally, as for the sequential ET, the evidence is conflicting: pooling of results of observational studies showed a significantly increased CPR while the results of the only included RCT demonstrated no benefit. Safety of this intervention is questionable. The transfer of two embryos at a distance and the transfer of the second one at the blastocyst stage may increase the risk of dizygotic and monozygotic twinning respectively^41^. Published data about these possible complications are reassuring but still insufficient. The potential serious obstetric and neonatal consequences and the unconvincing results on the efficacy discourage the conduct of further studies. Moreover, data demonstrating no differences in CPR for the first 6 IVF cycles deserve careful study on the role of chance and even of different multiple factors influencing CPR and LBR^178^.

Other treatment hypotheses might be valid and some RCTs are ongoing in order to test them. In this context, of particular relevance is the study protocol published by Lu et al.^179^. Authors aim to determine if prednisone can enhance live birth in women with RIF undergoing IVF. Interestingly, studies have shown that prednisone could not only suppress the inflammatory response in pre-implantation endometrium, but also stimulate the secretion of hCG and promote proliferation and invasion of trophoblast^179^. The efficacy of ad hoc treatments in women with known diseases and RIF also deserves to be clarified. In this context, the benefits and risks of aspirin and/or heparin in women with persistent antiphospholipid antibodies and RIF have been rather neglected until now.

### Strengths and limitations

To the best of our knowledge the present meta-analysis is the first to give a comprehensive view of the efficacy of all therapies or interventions proposed in order to improve IVF outcome in women with RIF. The population was selected according to strict inclusion criteria in order to reduce as much as possible the risk of misleading conclusions due to the high incidence of false positive diagnosis and, consequently, of inappropriate treatment. Moreover, being aware in advance of the limited available evidence, we decided to include also observational studies rather than limiting our analyses to RCTs. This choice allowed us also to also report on options that could become of interest in the future, i.e. once properly tested with RCTs.

Several limitations need to be considered in the interpretation of our results. First, many of the included studies suffered from serious risk of bias. Additionally, in the majority of cases, they recruited too few women to have enough statistical power to detect clinically relevant effect sizes, as is common in our field. Second, some studies included only frozen-thawed embryo replacement cycles while others only fresh IVF cycles. Furthermore, the protocols for ovarian stimulation, endometrial preparation, luteal phase support and the proposed interventions themselves also present marked variations between studies. In most cases, a proper investigation of this clinical heterogeneity was not feasible due to the limited number of studies. Third, in the present meta-analysis we focused on patients who had been investigated as much as possible to rule out possible known causes of RIF. However, it cannot be sustained with certainty that the selected population is affected by unexplained RIF. In fact, some contributions also included women of advanced age. In this context, it is pretty impossible to exclude the embryonic cause of RIF, without the use of PGT-A. Finally, there are few data addressing the safety profile of these treatments and their effect on the development and health of conceived children. Future studies focusing on treatment-related side effects and long-term follow-up data among the offspring are needed before introducing such interventions into daily clinical practice.

## Conclusion

In women with RIF, moderate quality evidence suggests that intrauterine PBMC infusion improves chances of clinical pregnancy and live birth and that subcutaneous G-CSF administration has a beneficial effect on CPR. These treatment options are the most promising among those investigated. However, prior to their introduction into routine clinical practice, high quality RCTs are needed. Trials design should include an identical placebo in the control arm to reduce performance bias and report ongoing pregnancy or live birth rate as primary outcome. The major and minor adverse effects of their administration should also be captured in any future studies.

Notably, our results should limit the use of many adjunct or add-on interventions in women with RIF whose prescription is currently extremely popular in IVF clinics around the world. In this regard, the administration of LMWH is not supported by evidence either regarding its efficacy or its safety profile. We also strongly discourage intentional endometrial injury with the aim of improving IVF outcome outside of registered experimental protocols.

RIF of unknown cause significantly hampers IVF success. An effective treatment strategy would constitute a revolution in the field. In this context, future research should focus on confirming therapeutic approaches for which robust efficacy data are already available [i.e. intrauterine PBMC infusion and subcutaneous G-CSF administration] before investigating new interventions or therapies or retest those supported by preliminary flabby evidence. Finally, regardless of the option to be tested, we plea for collaborative efforts that could allow to run large and robust RCTs. In recent years, RIF has become extremely popular with entire meetings exclusively dedicated to the argument. The time has now come for facts rather than speculations.
